# Work participation in inborn errors of immunity: The dual burden on patients and caregivers

**DOI:** 10.70962/jhi.20260077

**Published:** 2026-10-22

**Authors:** Federica Pulvirenti, Mayla Sgrulletti, Klara Radovic, Marco Lecis, Gloria Mantuano, Cinzia Milito, Alessandro Aiuti, Isabella Quinti, Viviana Moschese, AIP, Alessandro Segato, Filippo Cristoferi, Silvia Casati, ImmunITA, Pietro Andrea Accardo, Rosa Angarano, Federica Barzaghi, Caterina Cancrini, Clementina Canessa, Francesca Carfora, Maria Carrabba, Maria Pia Cicalese, Francesco Cinetto, Maria Giovanna Danieli, Giulia Di Colo, Giovanna Fabio, Andrea Finocchi, Giuliana Giardino, Giuseppe Ienopoli, Lorenzo Lodi, Baldassarre Martire, Silvia Ricci, Beatrice Rivalta, Riccardo Scarpa, Serena Serafino, Germano Sardella, Marta Chiara Sircana, Emanuele Vivarelli

**Affiliations:** 1 Reference Centre for Primary Immune Deficiencies, Sapienza University Hospital Policlinico Umberto I, Rome, Italy; 2 Pediatric Immunopathology and Allergology Unit, Policlinico Tor Vergata, University of Rome Tor Vergata, Rome, Italy; 3 Department of Transitional and Precision Medicine, Sapienza University of Rome, Viale dell’Università 37, Rome, Italy; 4 Department of Pediatrics, University of Bari “Aldo Moro,” Bari, Italy; 5 Department of Molecular Medicine, Sapienza University of Rome, Rome, Italy; 6 IRCCS San Raffaele Hospital and Vita-Salute San Raffaele University, Milan, Italy; 7 Sapienza Foundation, Sapienza University of Rome, Rome, Italy

## Abstract

Despite improved survival, occupational challenges in with inborn errors of immunity (IEI) patients and their families remain unmeasured. We conducted a nationwide cross-sectional survey promoted by AIP and ImmunITA, including 420 IEI adults and 110 caregivers, integrating occupational data with patient-reported outcome measures. Multivariable models assessed employment and productivity determinants. Overall, 67% of patients were employed, yet half reported reduced capacity, unsafe environments, limited career progression, and stigma. Unemployment was associated with female sex, disability, and lower education. Nondisclosure affected one-fourth of patients and one-fifth of caregivers. Work impairment reached 39%; absenteeism was driven by healthcare demands, while presenteeism was associated with disease burden, psychological distress, and immune dysregulation, but not the Ig administration route. Caregivers showed 34% work impairment, with absenteeism linked to care intensity and presenteeism to psychological distress, patient health, and child caregiving. Child caregivers had greater time demands and more part-time employment. In conclusion, IEI substantially impair work productivity, supporting integration of occupational needs into care to improve social inclusion.

## Teaser

Despite improved survival and workforce participation, IEI markedly impairs productivity in patients and caregivers, largely driven by presenteeism related to healthcare demands, psychological distress, inadequate workplace accommodations, and persistent stigma, highlighting a substantial and underrecognized clinical and societal burden.

## Graphical Abstract

**Figure:**
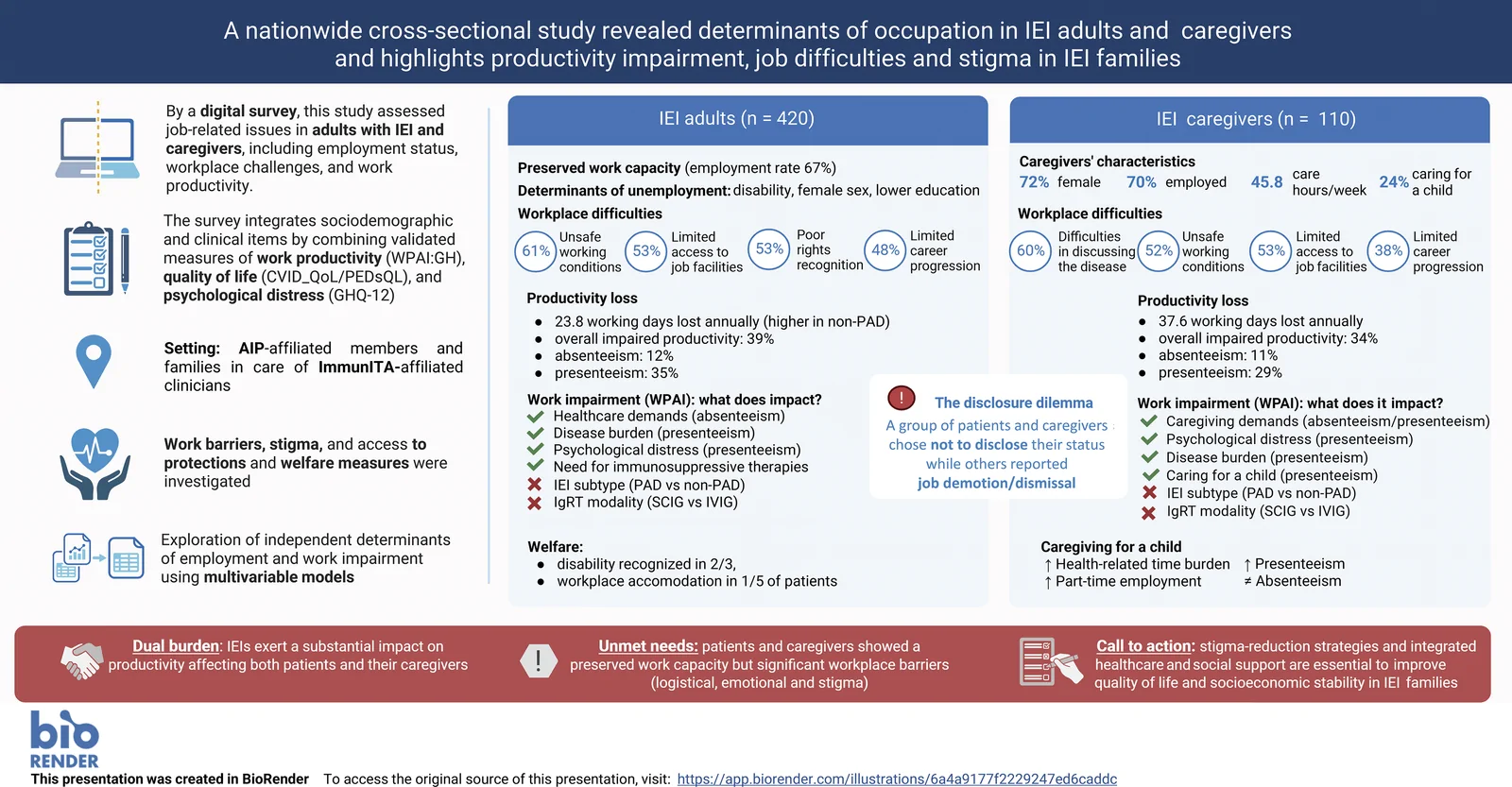

## Introduction

Inborn errors of immunity (IEI) are a heterogeneous group of >550 disorders characterized by intrinsic defects of the immune system, leading to increased susceptibility to recurrent infections, immune dysregulation, and malignancy (1). Although individually rare, IEI collectively represent chronic conditions requiring complex, lifelong management (2). The multiple clinical manifestations and physical dysfunctions associated with IEI require frequent healthcare visits and lifelong treatments, imposing a considerable burden on affected individuals and their daily functioning (3).

Advances in diagnostics, genetic testing, and the availability and use of Ig replacement therapy (IgRT), biologics, and small molecules, together with the expanded indications for hematopoietic stem cell transplantation (HSCT) and gene therapy (GT), have revolutionized the natural history of IEI (4, 5, 6, 7, 8, 9). As a result, a growing number of patients now reach adulthood and remain active in society and the workforce (10, 11). However, most of them still face medical complications, frequent hospitalizations, and treatment-related burdens (12), which impact physical and social activities and mental health (13, 14, 15, 16), ultimately affecting occupational outcomes and quality of life (17, 18, 19).

Health-related quality of life (HRQoL) is increasingly recognized as a key measure of well-being in chronic diseases and treatment effectiveness (20). Unlike traditional endpoints such as survival, HRQoL captures the broader impact of illness and its management on everyday life from the patient’s own perspective through validated patient-reported outcome measures (PROMs) (21). Several studies have demonstrated that disease-specific HRQoL instruments are particularly sensitive in detecting meaningful changes, providing a more accurate assessment of how treatments affect patients’ lived experiences compared with physicians’ evaluations (22). By focusing on functioning and well-being, HRQoL evaluation provides valuable guidance for therapeutic and management strategies (22, 23). Our group has previously investigated HRQoL in IEI, primarily focusing on common variable immunodeficiency (CVID) (15, 24, 25, 26, 27). Our validation of the CVID_QoL questionnaire (16), currently the only disease-specific HRQoL instrument available for IEI, demonstrated its utility in capturing disease burden and patient experience in IEI (28, 29, 30).

In adults, work participation is a key determinant of identity, well-being, and socioeconomic stability, and in chronic conditions it is strongly influenced by functional limitations, reduced HRQoL, and psychological distress (18, 31, 32, 33). However, evidence on employment patterns, work productivity, and workplace experiences in IEI remains limited (17, 34).

Likewise, the challenges faced by their informal caregivers—who often assume substantial emotional, logistical, and healthcare responsibilities—are largely unexplored, despite their central role in long-term disease management (35). Although caregiving may be associated with a sense of fulfilment, a high burden may be linked to adverse effects on physical and mental health, as well as social and professional life (36, 37). Accordingly, caregiver recognition and support have become key priorities for public health systems, and the World Health Organization advocates a dyadic approach that considers patients and caregivers as a single unit of care (38).

Understanding work-related outcomes in adults with IEI and their caregivers is essential to identify unmet needs that may disrupt work schedules and reduce productivity. Moreover, it remains unclear whether current health and social care systems adequately address these occupational challenges. To address this issue, we conducted a nationwide Italian study in collaboration with the Italian Association of IEI Patients (AIP) and the Italian Scientific Society of Immunodeficiencies (ImmunITA).

To our knowledge, this study is the first to evaluate occupational outcomes in a large IEI cohort by combining validated measures of productivity, disease-specific HRQoL, and psychological distress, and to assess the independent determinants of employment and work impairment. Moreover, this is the first study to assess work productivity among IEI caregivers.

With the integration of clinical information, PROMs, and socioeconomic indicators, we aimed to provide a comprehensive overview of work-related limitations in IEI patients and their caregivers, and to identify unmet needs to direct targeted healthcare and workplace strategies.

### Results

### Response rate and completeness of data

A total of 868 individuals were informed, and 631 questionnaires (73%) were returned; 101 were excluded (8 for lack of consent and 93 for incomplete employment data), resulting in a final sample of 530 participants.

### Characteristics of IEI adults

A total of 420 IEI adults were included in the analysis (62% women, median age 50 years, interquartile range [IQR]: 40–60). Over half were married (53%), and 87% cohabited with another. Most had at least a high school degree (89%). Median age at IEI symptom onset was 27 years (IQR: 10–42), and median age at diagnosis was 37 years (IQR: 25–50) (Table 1).

**Table 1.** Demographic characteristics of participants

Characteristic	IEI adults (*n* = 420)	IEI caregivers (*n* = 110)
Sex, female, *n* (%)	260 (62%)	79 (72%)
Age, median (IQR)	50 (40–60)	49 (41–59)
**Education, *n* (%)**	​	​
Primary school	5 (1%)	—
Middle school	64 (15%)	11 (10%)
High-school diploma	183 (44%)	51 (46%)
University degree or higher	168 (40%)	48 (44%)
**Family status, *n* (%)**	​	​
Single	113 (27%)	21 (19%)
Cohabiting	48 (11%)	12 (11%)
Married	224 (53%)	69 (63%)
Separated/divorced	26 (6%)	7 (6%)
Widowed	9 (2%)	1 (1%)
**Age at IEI onset, median (IQR)**	**27 (10–42)**	**NA**
**Age at IEI diagnosis, median (IQR)**	**37 (25–50)**	**NA**

NA, not available; IEI, inborn error of immunity; IQR, interquartile range.

**Figure 1.**
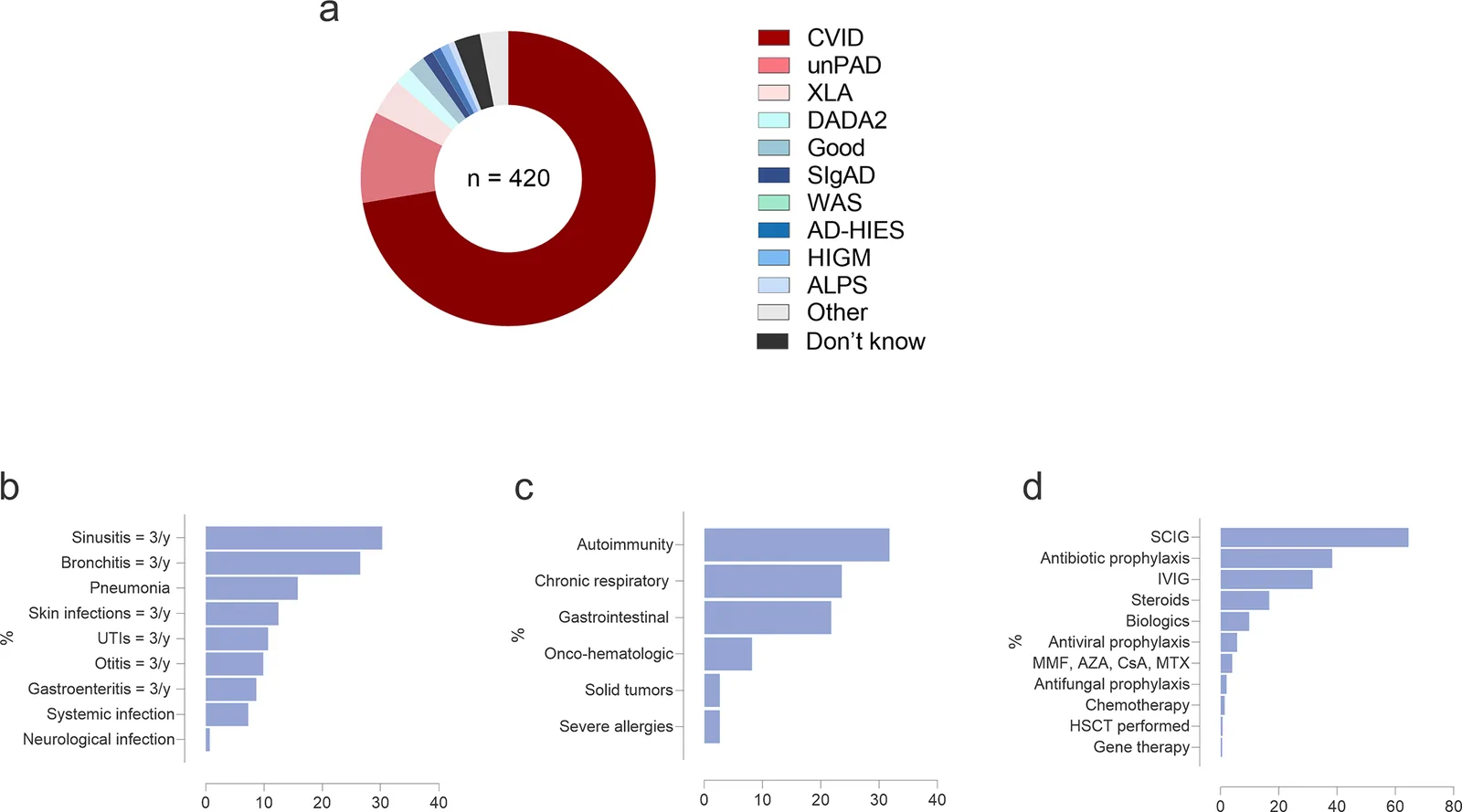
**Patients’ reported characteristics. (a–d)** Diagnosis (a); infections in the preceding year (b); noninfective IEI-related manifestations (c); treatments (d). AD-HIES, autosomal dominant hyper-IgE syndrome; ALPS, autoimmune lymphoproliferative syndrome; DADA2, deficiency of adenosine deaminase 2; Good, Good syndrome; HIGM syndrome, hyper-IgM syndrome; IEI, inborn error of immunty, SIgAD, selective IgA deficiency; unPAD, unclassified primary antibody deficiency; WAS, Wiskott–Aldrich syndrome; XLA, X-linked agammaglobulinemia; AZA, azathioprine; CsA, cyclosporine A; MMF, mofetil mycophenolate; MTX, methotrexate; UTIs, urinary tract infections; y, year.

According to the International Union of Immunological Societies (IUIS) classification (39), most patients were classified as predominantly antibody deficiencies (PAD; *n* = 372, 89%). Among the total cohort, CVID was the most frequent diagnosis (*n* = 306, 73%), followed by unclassified PAD (unPAD) (*n* = 38, 9%) and X-linked agammaglobulinemia (XLA) (*n* = 17, 4%), reflecting the predominance of antibody defects in adult European cohorts (10). Other IEI categories were represented by relatively small numbers, including primary immune regulatory disorders (*n* = 5, 1%), syndromic IEIs (*n* = 8, 2%), autoinflammatory diseases (ADA2 deficiency only, *n* = 9, 2%), phagocyte defects (*n* = 2, 1%), and phenocopies (Good syndrome only, *n* = 7, 2%). Seventeen patients were non-classified (4%). The complete list of diagnoses is reported in Fig. 1 a. A minority (*n* = 39, 9%) of participants reported an IEI-affected family member, who provided emotional (*n* = 26, 67%) and healthcare support (*n* = 9, 23%), cohabitation (*n* = 10, 26%), transport (*n* = 6, 15%), and financial help (*n* = 4, 10%).

Reported infections are summarized in Fig. 1 b. Most participants (*n* = 280, 67%) reported at least one IEI-related complication, including chronic lung disease (*n* = 139, 33%), gastrointestinal disorders (*n* = 126, 30%), autoimmune disease (*n* = 108, 26%), cancer (*n* = 30, 7%), and hematologic malignancies (*n* = 21, 5%) (Fig. 1 c). Treatments included IgRT (*n* = 373, 89%), mainly administered as subcutaneous Ig (SCIG) (*n* = 271, 64%). Additional antimicrobial therapies included microbial prophylaxis (antibiotics *n* = 161, 38%; antivirals *n* = 24, 6%; antifungals *n* = 9, 2%). One out of four patients (*n* = 107) presented noninfectious IEI complications requiring chronic immunosuppressive therapy, including steroids (*n* = 70, 17%), disease-modifying antirheumatic drugs (DMARDs, *n* = 17, 4%), and biologics (*n* = 41, 10%), with possible overlap between treatments. Chemotherapy was reported in a small proportion of patients (*n* = 6, 1%). HSCT and GT were reported in three and two patients, respectively (Fig. 1 d).

### Work participation in IEI adults

**Figure 2.**
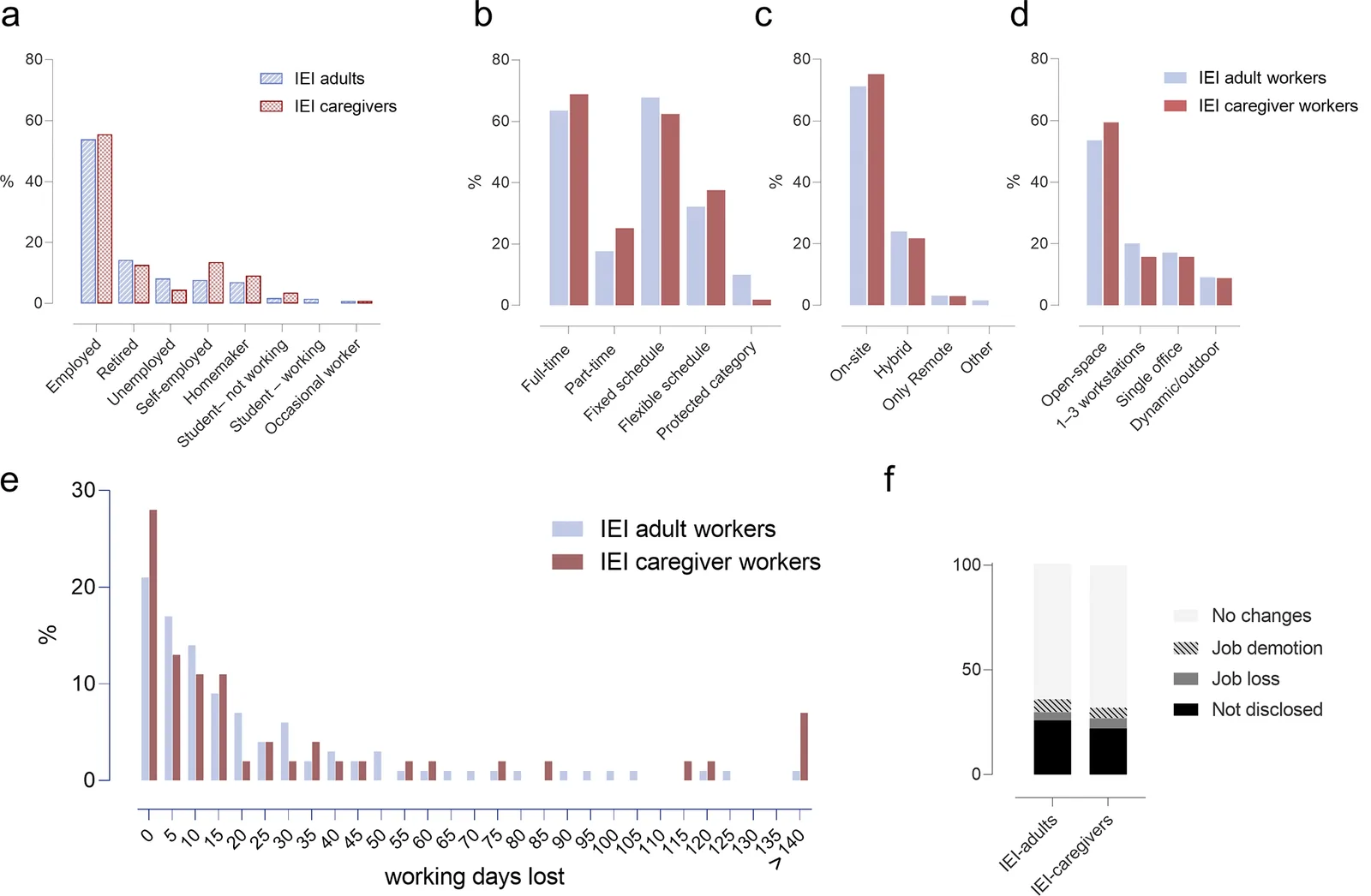
**Occupation in IEI adults and in IEI caregivers.** Job modalities in adults with IEIs (blue bars) and caregivers (red bars). **(a)** Employment status in 420 IEI adults and 110 caregivers. **(b–e)** Work characteristics in 282 employed IEI adults and 77 employed caregivers: work time arrangement (b), work modality (c), work setting (d), and distribution of working days lost in the previous year due to IEI-related health issues (e). **(f)** Disclosure of IEI diagnosis and its consequences in 402 IEI adults and 107 caregivers with previous work experience. IEI, inborn error of immunity.

**Figure S1.**
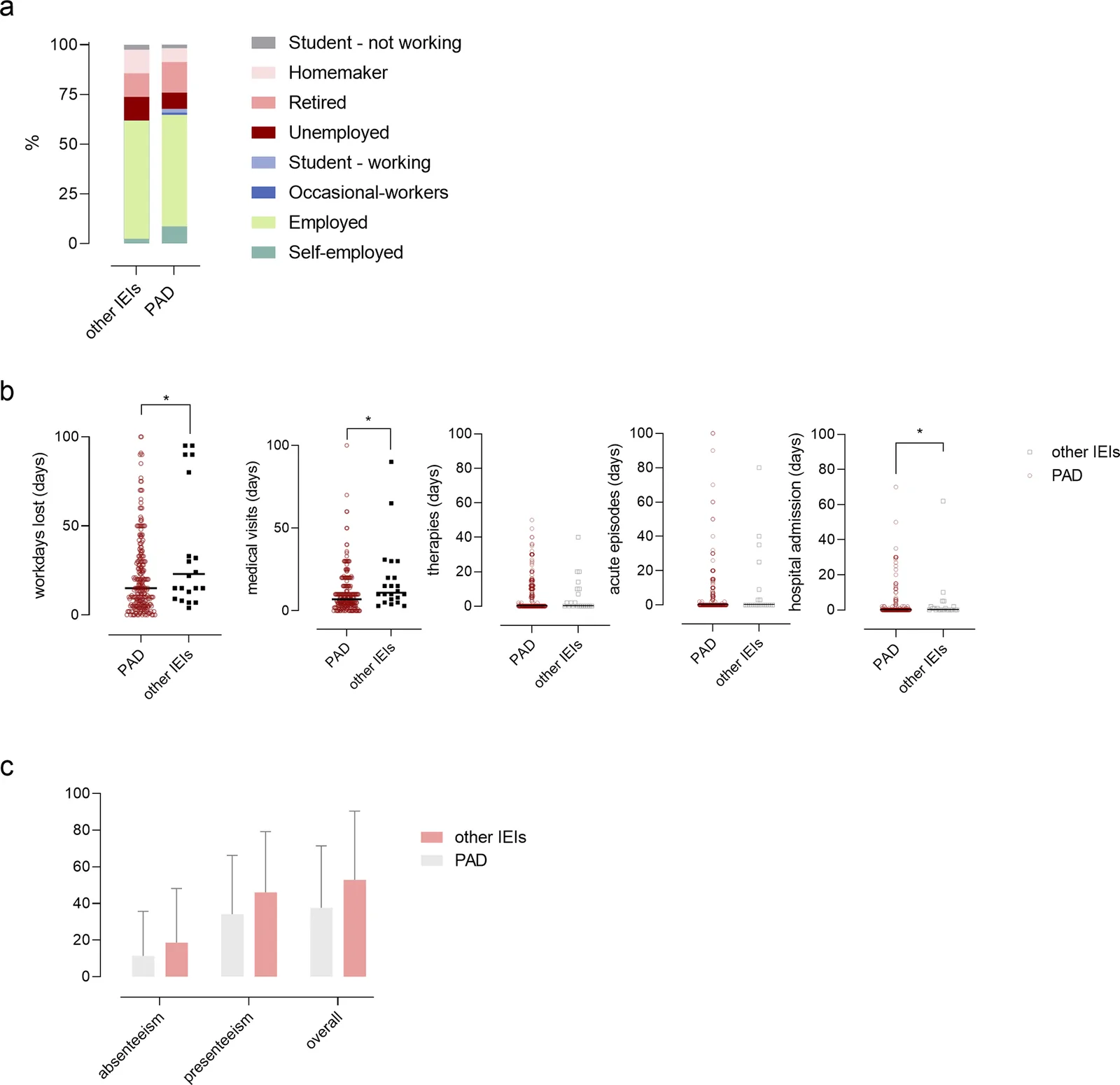
**Occupation and work productivity and activity impairment across IEI categories. (a)** Distribution of employment status in PAD (*n* = 372) vs. other IEIs (*n* = 48). **(b)** Days lost due to IEI-related health issues, including total workdays lost, medical visits, therapies, acute episodes, and hospitalizations, comparing PAD and other IEIs. Bars represent means. *P < 0.05 (Mann–Whitney U test). **(c)** Values of absenteeism, presenteeism, and overall work impairment across PAD and other IEIs. Bars represent mean (±SD). No statistically significant differences were observed across groups (Mann–Whitney U test).

96% of IEI adults (*n* = 402) reported previous work experience. At the study time, 67% (*n* = 282) were employed, 15% (*n* = 63) were retired, and 18% (*n* = 75) were not employed. Employed participants were predominantly dependent workers (*n* = 237, 56%), followed by self-employed (*n* = 34, 8%), with smaller proportions of occasional workers (*n* = 4, 1%) and working students (*n* = 7, 2%) (Fig. 2 a). Employment distribution was similar between PAD and other IEIs (Fig. S1 a).

Most employed individuals worked full-time (*n* = 201, 71%), on-site (*n* = 177, 63%), and in open-space environments (*n* = 151, 53%), while 20% (*n* = 57) worked in small work settings with 1–3 colleagues, 15% (*n* = 43) in single offices, and 33% worked remotely, either fully (*n* = 12, 4%) or in a hybrid setting (*n* = 80, 28%) (Fig. 2, b–d). Results of the univariate analysis revealed factors associated with employment as reported in Table S2. Employment status was associated with higher educational level (odds ratio [OR]: 8.63, P < 0.0001), lower disability burden (OR: 0.19, P < 0.0001), and fewer IEI-related complications (OR: 0.47, P = 0.033), as well as younger age at study time (P < 0.0001), younger age at IEI diagnosis (P = 0.006), and better HRQoL (CVID_QoL total, P = 0.010). In contrast, IEI diagnosis (PAD vs. other IEIs), treatment modality at the study time (intravenous Ig [IVIG] vs. subcutaneous Ig [SCIG]), and the presence of other affected family members were not associated (P > 0.05). In the multivariable model, female sex (OR: 0.22, P = 0.006) and moderate-to-severe disability (OR: 0.17, P = 0.001) were independently associated with a lower likelihood of being employed, whereas higher educational level was associated with a higher likelihood of employment (OR: 7.40, P < 0.0001), while age at study time, age at IEI diagnosis, IEI subtype (PAD vs. other IEIs), IEI-related complications, and HRQoL at the study time were not associated with employment status (Table 2). No evidence of multicollinearity was observed among the variables included in the model (all variance inflation factors [VIFs] <2.6, Table S3).

**Table 2.** Multivariable logistic regression analysis of factors associated with employment in IEI adults

Variable	OR (95% CI)	P value
Age	0.97 (0.91–1.03)	0.362
Female sex	0.22 (0.07–0.66)	0.006
Higher education	7.40 (2.59–21.18)	<0.0001
PAD (vs. other IEIs)	0.85 (0.17–4.16)	0.845
IEI-related complications	0.35 (0.08–1.49)	0.154
Disability (moderate/severe)	0.17 (0.06–0.46)	0.001
Age at IEI diagnosis	0.99 (0.94–1.03)	0.609
CVID_QoL total	0.99 (0.96–1.01)	0.386

CVID_QoL, common variable immunodeficiency quality of life questionnaire; IEI, inborn error of immunity; OR, odds ratio; CI, confidence interval; PAD, predominantly antibody deficiency.

**Figure 3.**
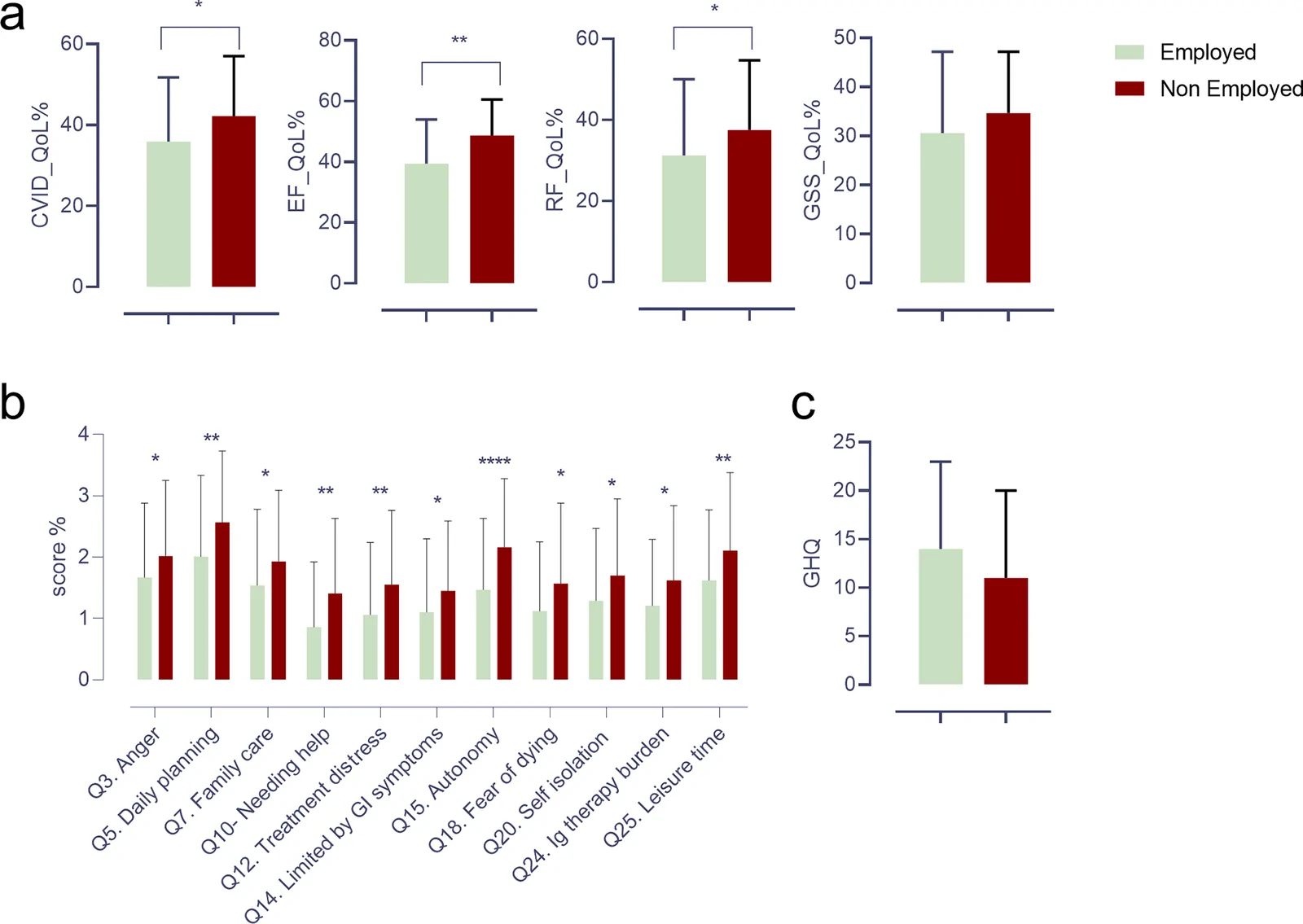
**HRQoL in employed vs. non-employed. (a–c)** CVID_QoL total score and domains (a), individual CVID_QoL items (b), and GHQ scores (c) in employed vs. non-employed IEI patients. Only items showing significant between-group differences are presented; full results are reported in Table S4. P values were calculated using the Mann–Whitney U test. Significance levels: *P < 0.05, **P < 0.01, and ****P < 0.0001. CVID_QoL, common variable immunodeficiency quality of life; GSS, gastrointestinal and skin symptoms; IEI, inborn error of immunity; Q, question; GHQ, general health questionnaire.

Compared with employed individuals, non-employed IEI adults showed worse HRQoL across multiple domains, including CVID_QoL total (P = 0.010), emotional functioning (EF) (P = 0.007), and relational functioning (RF) (P = 0.035), along with higher impairment in autonomy, daily planning, leisure activities, family care, social support, illness-related fears, and treatment burden (all P < 0.05). However, psychological distress did not differ between groups (Fig. 3, a–c, and Table S4).

### Work-related difficulties in IEI workers

Employed IEI patients lost a mean of 23.8 (SD: 29.9) working days annually due to IEI-related health issues, with a highly skewed distribution (median 15 days, IQR: 6–33). The distribution of work loss was heterogeneous, with 30% of individuals reporting >20 days lost, followed by 21% losing 11–20 days, 19.0% losing 6–10 days, and 26% losing 5 or fewer days per year (Fig. 2 e). This burden was attributable to 11.4 days spent on medical visits or examinations (SD: 13.5), 6.3 days for treatment administration or adverse events (SD: 11.4), 5.5 days due to acute episodes (SD: 15.0), and 2.7 days due to hospitalizations (SD: 8.7). When comparing PAD vs. other IEIs, non-PAD patients showed a higher burden of work loss, with significantly more days dedicated to medical visits and examinations (P = 0.023) and hospitalizations (P = 0.010), resulting in a higher total number of workdays lost (P = 0.049). No differences were observed for days lost due to therapies or acute episodes (Fig. S1 b).

**Figure 4.**
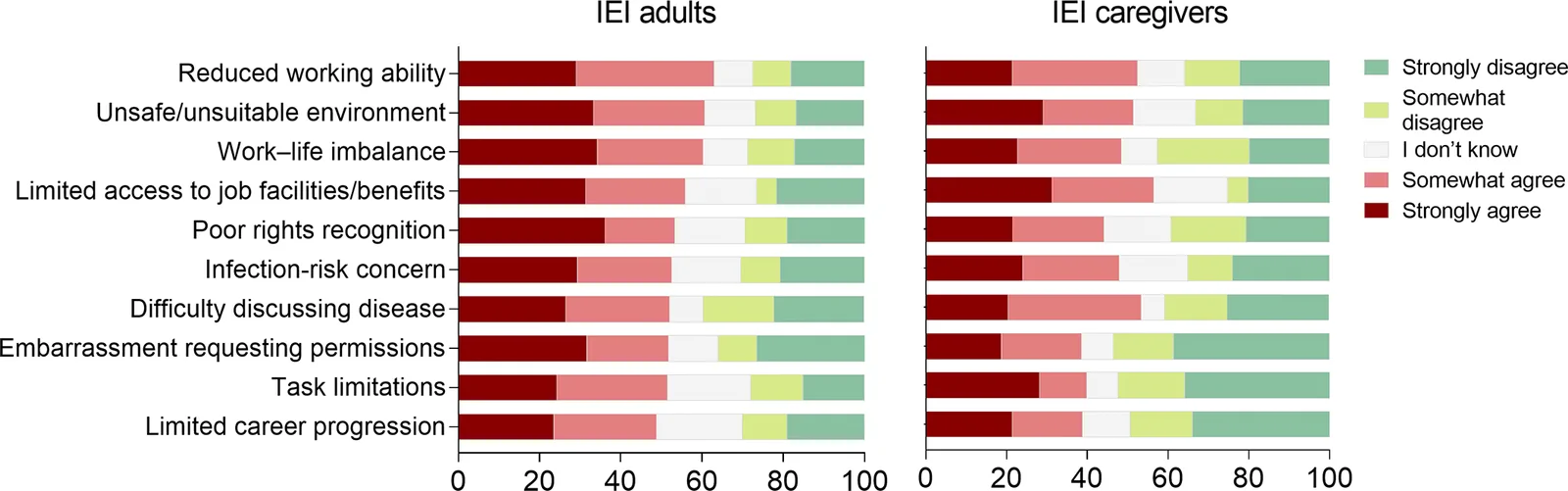
**Work-related difficulties in IEI workers and IEI caregivers.** Stacked bar charts show responses to survey items on job-related difficulties using a five-point Likert scale (questionnaire content shown in Table S1). IEI, inborn error of immunity.

Workplace difficulties were common. Nondisclosure of the IEI diagnosis at the workplace was reported in 24% of patients with work experience; after disclosure, 9% reported job demotion and 5% reported job loss (Fig. 2 f). Reduced working ability was reported by 63%, task limitations by 51.5%, and negative impact on career opportunities by 49%. Environmental barriers included unsafe or unsuitable workplaces (61%). More than half of respondents reported limited access to job benefits (56%) and poor recognition of their rights (53%). Insufficient attention from colleagues to infection risk was reported by 53%, alongside difficulties discussing the disease at work (52%) and embarrassment when requesting leave (52%) (Fig. 4).

### Work productivity and activity impairment in IEI adults

In employed IEI adults, work productivity loss, as assessed by the Work Productivity and Activity Impairment: General Health (WPAI:GH), corresponded to a mean of 4.3 h (SD: 14.6) in the previous week. Overall, they missed 12% (SD: 25%) of their working time due to absenteeism and reported an additional 35% (SD: 32%) reduction in productivity while working, resulting in 39% overall work impairment (SD: 34%) (Table 3).

**Table 3.** Work productivity and activity impairment of employed adults with IEI and caregivers

WPAI:GH Parameters	Employed IEI adults (*n* = 282)	Employed IEI caregivers (*n* = 77)
Absenteeism (% work time missed), mean (SD)	12.0 (24.8)	10.8 (21.8)
Presenteeism (% impairment while working), mean (SD)	35.1 (32.4)	29.4 (30.0)
Overall work impairment (%), mean (SD)	39.0 (34.3)	34.2 (32.0)
Activity impairment (%), mean (SD)	34.9 (33.3)	18.7 (32.3)

WPAI:GH, Work Productivity and Activity Impairment: General Health; IEI, inborn error of immunity; SD, standard deviation.

In univariable analyses, WPAI domains were consistently associated with markers of disease burden and healthcare-related time, including CVID_QoL scores, psychological distress (general health questionnaire [GHQ]), time spent on medical visits and treatments, together with infections per year, IEI-related complications, and the use of immunosuppressive treatments. In contrast, sociodemographic factors such as age, sex, and educational level, diagnostic delay, a diagnosis of non-PAD IEI, and IgRT modality showed no consistent associations (Table S5). WPAI domains across PAD vs. other IEIs are shown in Fig. S1 c.

In multivariable analyses including IEI diagnosis, clinical, treatment-related, and sociodemographic variables, overall work impairment was independently associated with disease burden (CVID_QoL: B = 0.70, P < 0.0001), psychological distress (positive screening for psychological distress [GHQ+]: B = 11.47, P = 0.010), time spent on healthcare visits (B = 0.52, P < 0.0001), and the presence of noninfective IEI manifestations requiring immunosuppressive therapy (B = 15.20, P < 0.0001). Absenteeism was mainly associated with healthcare-related time burden, particularly days spent on visits (B = 0.69, P < 0.0001), together with disease burden (CVID_QoL: B = 0.22, P = 0.047). Similarly, presenteeism was associated with disease burden (CVID_QoL: B = 0.73, P < 0.0001) and healthcare-related time (visits: B = 0.44, P = 0.001) but also with psychological distress (GHQ+: B = 11.22, P = 0.007) and the presence of noninfective IEI manifestations requiring immunosuppressive therapy (B = 11.62, P = 0.003). Finally, activity impairment outside the workplace was associated with disease burden (CVID_QoL: B = 0.96, P < 0.0001), psychological distress (GHQ+: B = 11.12, P = 0.016), and the presence of noninfective IEI manifestations requiring immunosuppressive therapy (B = 12.85, P = 0.003) and was higher in women (B = 7.74; P = 0.033). No significant associations were observed for non-PAD diagnosis and IgRT modality (SCIG vs. IVIG) across WPAI domains. All models showed low multicollinearity (VIF <2, Table S6).

### Characteristics of IEI caregivers

A total of 110 caregivers participated in the survey (72% female; median age 49 years, IQR: 41–59). Most had a high school degree or higher (*n* = 99, 90%) and were married/cohabiting (*n* = 81, 74%). The median age at the time of the relative’s diagnosis was 39 years (IQR: 30–48) (Table 1).

**Figure S2.**
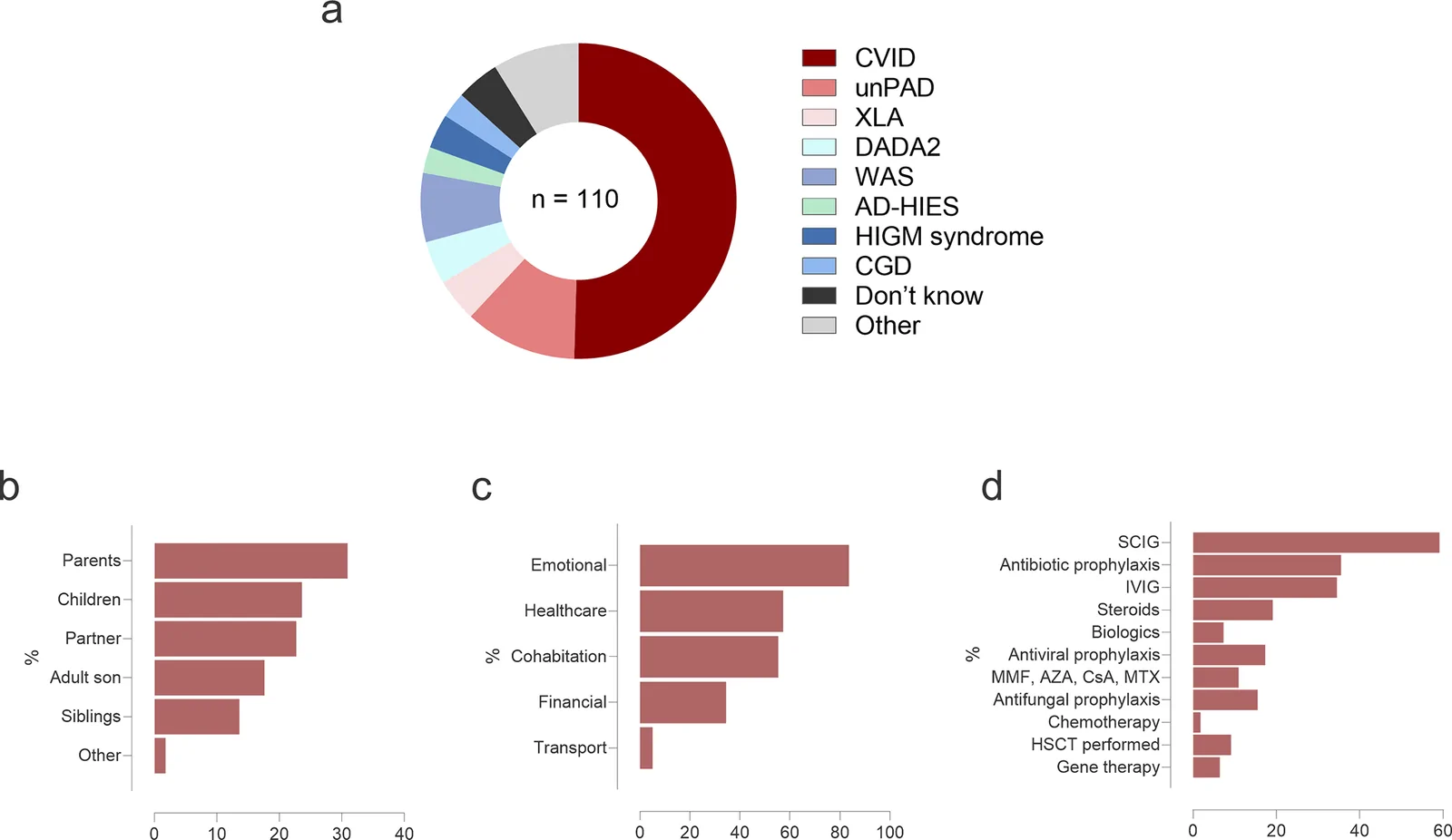
**Reported characteristics of patients cared by caregivers. (a–d)** IEI diagnosis (a); relationship with caregivers (b); caregivers’ duties (c); and treatments (d). AD-HIES, autosomal dominant hyper-IgE syndrome; ALPS, autoimmune lymphoproliferative syndrome; DADA2, deficiency of adenosine deaminase 2; Good, Good sybdrime; HIGM, hyper-IgM syndrome; IEI, inborn error of immunty; SIgAD, selective IgA deficiency; unPAD, unclassified primary antibody deficiency; WAS, Wiskott–Aldrich syndrome; XLA, X-linked agammaglobulinemia; AZA, azathioprine; CsA, cyclosporine A; MMF, mofetil mycophenolate; MTX, methotrexate; UTIs, urinary tract infections; y, year.

According to the IUIS classification (39), most patients assisted by caregivers were classified as PAD (*n* = 79, 72%): CVID was the most frequent diagnosis (*n* = 56, 51%), followed by unPAD (*n* = 13, 12%) and XLA (*n* = 6, 5%) (Fig. S2 a). Twenty-four percent of caregivers cared for children aged <18 years (*n* = 26), while the majority cared for adult individuals, including parents (*n* = 34, 31%), partners (*n* = 25, 23%), young adults (*n* = 23, 21%), siblings (*n* = 15, 14%), or others (*n* = 2, 2%) (Fig. S2 b). Patients’ median age at diagnosis was 22 years (IQR: 5–42), and one-third were diagnosed within 10 years. Among caregivers of pediatric patients, care recipients were predominantly adolescents (30% aged 13–18 years), followed by children 0–4 years (25%), 5–7 years (25%), and 8–12 years (20%).

Caregivers dedicated a mean of 45.8 (SD: 58.4) hours/week (median 20, IQR: 3–70) to caregiving tasks, including emotional support (*n* = 92, 84%), healthcare-related (*n* = 63, 58%), transportation (*n* = 57, 52%), and financial support (*n* = 38, 34%). More than half of caregivers (*n* = 61, 55%) cohabited with the patient (Fig. S2 c). Notably, caregivers of children reported a significantly higher weekly caregiving burden (median 55 vs. 14 h, P = 0.024). Most patients were reported to have received IgRT (*n* = 98, 89%), often subcutaneously (*n* = 61, 62%), along with antimicrobial prophylaxis (antibiotics *n* = 39, 35%, antivirals *n* = 19, 17%, antifungals *n* = 17, 15%). 23% of patients (*n* = 25) presented with noninfectious IEI complications requiring chronic immunosuppressive therapy, including steroids (*n* = 21, 19%), DMARDs (*n* = 12, 11%), and biologics (*n* = 8, 7%), with possible overlap between treatments. Chemotherapy was reported in 2% of patients (*n* = 2), while HSCT and GT were reported in 9% (*n* = 10) and 6% (*n* = 7) (Fig. S2 d).

### Work participation and difficulties in IEI caregivers

Almost all caregivers included in the study (97%, *n* = 107) reported previous work experience. At the time of the survey, 70% (*n* = 77) were employed, including 55% (*n* = 61) working as employees, 14% (*n* = 15) as self-employed, and 1% (*n* = 1) as a seasonal worker. A smaller proportion were homemakers (*n* = 10, 9%), unemployed (*n* = 5, 5%), retired (*n* = 14, 13%), or non-working students (*n* = 4, 4%) (Fig. 2 a). Working caregivers were predominantly full-time (*n* = 51, 66%), on-site (*n* = 49, 63%), and with fixed schedules (*n* = 44, 57%). Caregivers assisting children were more likely to work part-time compared with those caring for adults (OR: 3.13, 95% confidence interval [CI]: 1.03–9.54; P = 0.041). Only a minority of IEI caregivers worked in hybrid mode (*n* = 21, 27%) or exclusively remotely (*n* = 3, 4%). Most worked in shared open-space settings (*n* = 43, 55%), whereas smaller proportions worked in small offices with 1–3 colleagues (*n* = 12, 16%), in single offices (*n* = 11, 14%), or in dynamic work environments (*n* = 7, 9%) (Fig. 2, b–d). Higher educational level was the only factor associated with employment (P < 0.001; OR: 18.75), while clinical characteristics, non-PAD diagnosis, IgRT modalities, or noninfective manifestations requiring immunosuppressive therapy showed no significant associations (Table S7).

Caregivers reported a mean loss of 37.6 working days/year (SD: 79.2), with a markedly skewed distribution (median 9 days, IQR: 1–38). The distribution of work loss was highly heterogeneous and mirrored that reported by patients, with 32% of caregivers reporting >20 days lost, followed by 14% losing 11–20 days, 17% losing 6–10 days, and 38% losing 5 or fewer days annually (Fig. 2 e). This burden was primarily driven by hospitalizations, accounting for a mean of 17.2 days (SD: 31.4), followed by medical visits and examinations (13.3 days, SD: 19.9), therapies or treatment-related adverse events (10.9 days, SD: 22.7), and the management of acute episodes (9.7 days, SD: 23.5), all showing substantial variability. When comparing caregivers of PAD vs. non-PAD patients, a significantly higher burden of healthcare-related time was observed in the non-PAD group, including more days spent on medical visits (P = 0.001), therapies (P = 0.032), and hospitalizations (P = 0.001). In a complementary analysis, caregivers of pediatric patients also reported a higher burden of care, particularly in terms of hospitalizations (P = 0.041), compared with those caring for adults.

Work difficulties included embarrassment when requesting leave (56%), limited access to workplace benefits (51%), unsafe environment (51%), insufficient attention from colleagues to infection risks (48%), difficulties communicating caregiving responsibility or reduced work ability because of family responsibilities (47%), difficulties in maintaining work–life balance (44%), and difficulty obtaining formal recognition of caregiver rights (41%). Limited career opportunities and task modifications due to caregiving status were reported in 39% and 37%, respectively (Fig. 4). Notably, among caregivers with previous work experience, 22% reported not disclosing their caregiving status, while job loss and demotion were each reported by 6% following disclosure (Fig. 2 f).

### Work productivity in IEI caregivers

Using the WPAI questionnaire, caregivers were found to miss, on average, 11% (SD: 22%) of their working time due to absenteeism and reported a 29% (SD: 30%) reduction in on-the-job productivity (presenteeism) (Table 3).

The results of univariate analyses assessing factors associated with WPAI outcomes are reported in Table S5. WPAI domains were consistently associated with markers of disease burden and healthcare-related time, including CVID_QoL scores, psychological distress (GHQ+), and time spent on caregiving, especially for visits and treatments. In contrast, sociodemographic factors, diagnostic delay, IEI manifestations requiring immunosuppressive therapy, or IgRT modality showed no consistent associations across domains, whereas absenteeism was higher in those diagnosed with a non-PAD IEI (P = 0.006).

In domain-specific multivariable models (Table 4), absenteeism was associated with caregiving intensity (weekly hours of care: B [regression coefficient] = 0.094, P = 0.016), whereas presenteeism was independently associated with psychological distress (GHQ+ B = 15.129, P = 0.019) and poorer patient health status (pediatric quality of life inventory [PedsQL] B = −0.485, P = 0.001), and was higher among caregivers assisting a child with IEI (B = 14.679, P = 0.044). Overall work impairment was primarily explained by patient health status (PedsQL B = −0.420, P = 0.017). In contrast, activity impairment outside the workplace was linked to the patient’s disability level (moderate-to-severe B = 37.598, P = 0.038), suggesting a differential impact of clinical and psychosocial factors across the productivity domain (Table 4). Multicollinearity diagnostics showed all VIF values below two across all WPAI models (Table S6).

**Table 4.** Multivariable determinants of WPAI domains in IEI adults and caregivers

Variable	Absenteeism B (95% CI)	P value	Presenteeism B (95% CI)	P value	Overall work impairment B (95% CI)	P value	Activity impairment B (95% CI)	P value
**IEI adults**	​	​	​	​	​	​	​	​
CVID QoL score	**0.22 (0.00 to 0.44)**	**0.047**	**0.73 (0.50 to 0.97)**	**<0.001**	**0.70 (0.45 to 0.95)**	**<0.001**	**0.96 (0.71 to 1.21)**	**<0.001**
GHQ+	1.43 (−6.18 to 9.04)	0.712	**11.22 (3.06 to 19.39)**	**0.007**	**11.47 (2.75 to 20.20)**	**0.010**	**11.12 (2.12 to 20.13)**	**0.016**
Days for visits/exams	**0.69 (0.45 to 0.92)**	**<0.001**	**0.44 (0.20 to 0.69)**	**0.001**	**0.52 (0.26 to 0.79)**	**<0.001**	−0.00 (−0.30 to 0.30)	0.985
Days for therapies	0.16 (−0.10 to 0.42)	0.216	−0.00 (−0.28 to 0.28)	0.986	0.04 (−0.26 to 0.33)	0.812	−0.08 (−0.36 to 0.20)	0.562
Age	−0.10 (−0.36 to 0.17)	0.487	−0.26 (−0.55 to 0.03)	0.078	−0.30 (−0.61 to 0.01)	0.058	0.22 (−0.08 to 0.52)	0.154
Sex (female)	−0.01 (−6.07 to 6.05)	0.998	2.93 (−3.57 to 9.43)	0.375	2.71 (−4.24 to 9.66)	0.443	**7.74 (0.64 to 14.84)**	**0.033**
IEI diagnosis (PAD vs. others)	3.35 (−8.10 to 14.80)	0.565	0.59 (−11.74 to 12.92)	0.925	0.62 (−12.56 to 13.79)	0.927	−0.52 (−14.17 to 13.14)	0.941
IgRT (SCIG vs. IVIG)	−3.73 (−10.37 to 2.91)	0.270	−1.83 (−8.98 to 5.31)	0.614	−2.33 (−9.96 to 5.31)	0.549	−1.33 (−9.04 to 6.39)	0.735
Immunosuppressive therapy	5.22 (−1.81 to 12.25)	0.145	**11.62 (4.06 to 19.19)**	**0.003**	**15.20 (7.11 to 23.29)**	**<0.001**	**12.85 (4.41 to 21.29)**	**0.003**
**IEI caregivers**	​	​	​	​	​	​	​	​
Age	−0.1 (−0.5 to 0.3)	0.663	−0.1 (−0.7 to 0.5)	0.799	−0.2 (−0.9 to 0.5)	0.581	—	—
Caring for a child	3.3 (−6.3 to 12.9)	0.492	**14.7 (0.4 to 29.0)**	**0.044**	12.7 (−3.8 to 29.2)	0.128	—	—
PedsQL total score	−0.1 (−0.3 to 0.1)	0.524	**−0.5 (−0.8 to −0.2)**	**0.001**	**−0.4 (−0.8 to −0.1)**	**0.017**	—	—
GHQ+	5.5 (−2.7 to 13.8)	0.183	**15.1 (2.6 to 27.7)**	**0.019**	14.2 (−0.8 to 29.2)	0.062	15.0 (−16.8 to 46.8)	0.301
Weekly hours of care	**0.1 (0.0 to 0.2)**	**0.016**	0.0 (−0.1 to 0.1)	0.467	0.1 (−0.1 to 0.2)	0.361	0.0 (−0.2 to 0.2)	0.757
Patient disability (moderate/severe)	—	—	—	—	—	—	**37.6 (2.8 to 72.4)**	**0.038**

Bold values indicate statistically significant results (P < 0.05). CVID_QoL, common variable immunodeficiency quality of life questionnaire; GHQ, general health questionnaire; GHQ+, positive screening for psychological distress; IEI, inborn error of immunity; IgRT, immunoglobulin replacement therapy; IVIG, intravenous immunoglobulin; PAD, predominantly antibody deficiency; PedsQL, pediatric quality of life inventory; SCIG, subcutaneous immunoglobulin; WPAI, work productivity and activity impairment; B, regression coefficient; CI, confidence interval.

### Work-related disability certifications

**Figure S3.**
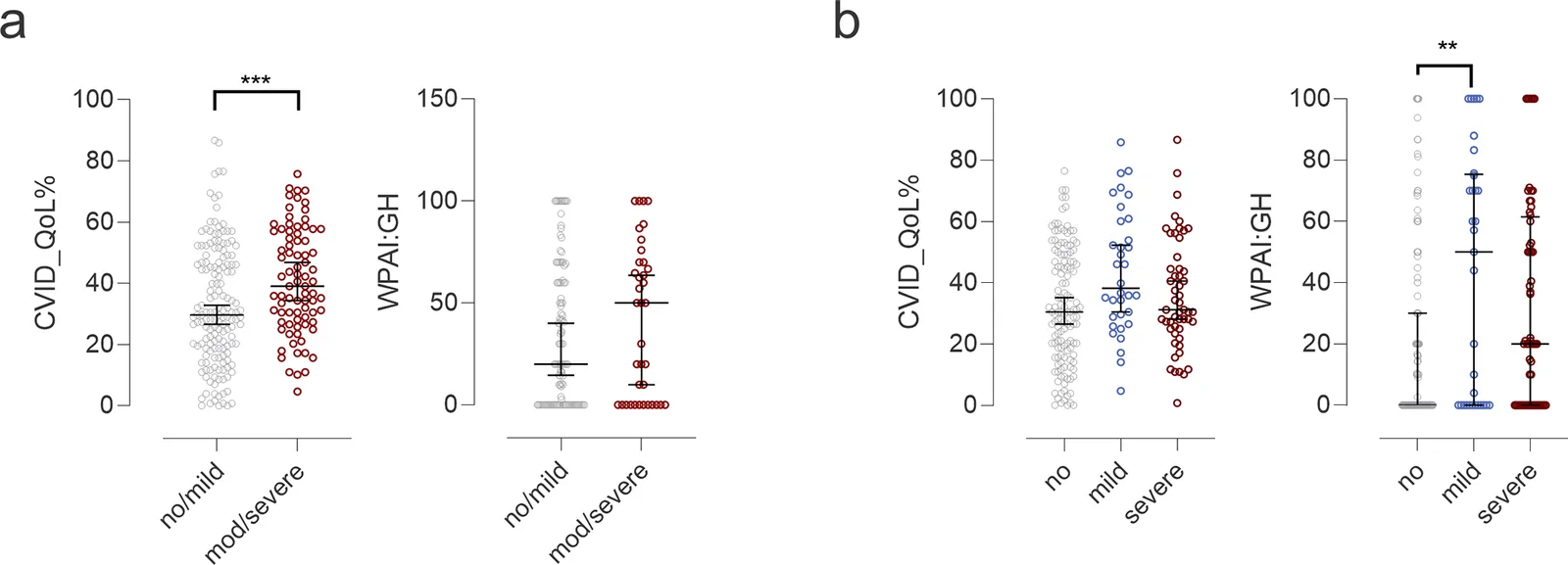
**Disability certification and occupational burden. (a and b)** CVID_QoL and WPAI:GH scores stratified by disability severity (no/mild <67% vs. moderate [mod]/severe ≥67%) (a) and daily functioning impairment (none, mild, and severe) (b). P values were calculated using the Mann–Whitney U test. Levels of significance: ***P < 0.001; **P < 0.01.

In Italy, disability status is assessed and certified by the National Institute for Social Security (INPS), which assigns a percentage of disability based on functional impairment and its impact on daily activities (40). Overall, 69% of IEI adults reported a formally recognized disability rating, with 35% classified as moderate/severe (67–99%) and 12% were classified as 100% disabled. Furthermore, 14% of patients were employed under protected employment categories, a system to facilitate workforce inclusion through targeted placement for those with certified disabilities ≥46% (41). In addition to disability certification, Italian welfare provides social protection for individuals with severe impairments affecting daily functioning and autonomy, including workplace accommodations, protected leave, and specific caregiver-related benefits (42). In our cohort, a severe functioning impairment was certified in only 21% of participants (Table 5). Notably, disability rating or functional impairment status was not associated with WPAI and CVID_QoL outcomes (Fig. S3), suggesting that current systems may not adequately capture the real-world occupational and disease burden of IEI.

**Table 5.** Disability status, legal frameworks, and associated benefits assigned to IEI patients and caregivers

Framework	Category	IEI patients, *n* (%)	IEI caregivers, *n* (%)	Functional meaning	Main benefits
Disability rating (INPS)	None	112 (28.0)	16 (14.4)	No certified disability	No entitlement to disability-related benefits
	Minimal (<33%)	8 (2.0)	5 (4.5)	No relevant functional limitation	No entitlement to specific benefits or allowances
	Mild (34–45%)	17 (4.0)	0 (0.0)	Recognized impairment	Access to prescribed medical aids (e.g., mobility, respiratory, and hearing devices) reimbursed by the National Health Service after specialist evaluation
	Protected employment (46–66%)	66 (15.7)	18 (16.4)	Work-limiting condition	Targeted employment placement through reserved job quotas, priority hiring, and workplace accommodation, including task modification and flexible arrangements (art. 18, Law *n*. 68/1999)
	Moderate (67–73%)	55 (13.1)	12 (10.9)	Clinically relevant disability	Healthcare copayment exemption, access to disability-related financial support, social assistance services, and income-dependent allowances
	Severe (74–99%)	94 (22.4)	16 (14.5)	Significant functional impairment	Monthly disability allowance, income-based, with access to additional social support measures and services
	Total (100%)	52 (12.4)	43 (39.1)	Complete inability to work	Disability pension, with possible attendance allowance and access to comprehensive social and healthcare support services
	Unknown	18 (4.3)	0 (0.0)	—	Not specified
Daily functioning/autonomy (Law 104/1992)	None	219 (52.1)	43 (39.1)	No impairment	No entitlement to Law 104–related benefits
	Assigned, severity not specified	31 (7.4)	12 (10.9)	Functional limitations not classified	—
	Mild impairment	56 (13.3)	4 (3.6)	Non-severe limitation	Workplace accommodations, tax benefits, and eligibility for flexible work arrangements
	Severe impairment	90 (21.4)	51 (46.4)	Severe limitations in autonomy	Paid leave (3 days/mo), caregiver leave, flexible work arrangements, priority job transfer, and tax benefits
	Unknown	53 (12.6)	0 (0.0)	—	Not specified

Disability rating and impairment in daily functioning/autonomy were assessed and certified by the INPS. Workplace accommodations and related protections are regulated by Italian Law No. 104/1992 (43).

## Discussion

The life-long nature of IEI still poses major challenges to patients’ lives despite advances in diagnosis and therapy. Over recent decades, survival in patients with IEI has substantially improved. As a result, more individuals are reaching adulthood (44, 45, 46). IEIs are now increasingly managed as chronic conditions, drawing attention to their impact on daily functioning, including employment and work productivity.

Due to the immune defect, individuals with IEI present specific and often under-recognized workplace needs. These include minimizing exposure to infectious risks, ensuring adequate recovery time following treatments, and providing flexibility to attend scheduled medical visits, which are essential to prevent complications or enable early intervention. However, evidence on how impaired HRQoL translates into real-world employment outcomes remains limited.

To our knowledge, this is the largest study to evaluate occupational outcomes in IEI, and the first to systematically assess caregiver work productivity. By integrating multiple validated PROMs, we provided a comprehensive evaluation of the clinical and psychosocial determinants of work impairment and identified independent factors associated with employment and productivity, offering actionable targets for clinical and policy interventions (Video 1).

**Video 1:** **Work participation and productivity in adults** **with inborn errors of immunity and their caregivers.** Video summary of a nationwide cross-sectional digital survey illustrating the impact of IEIs on employment, work productivity, and workplace challenges among adults with IEI and their informal caregivers. The video highlights the main determinants of work impairment and potential interventions to support work participation among adults with IEI and their families.

Most Italian IEI adults showed preserved work capacity, with an employment rate of 67%, in line with the Italian general population (47), slightly higher than that reported in a smaller Japanese IEI cohort (17). Despite this, workplace difficulties were common, with many patients reporting reduced work capacity, limited career opportunities, embarrassment in requesting leave, or unsafe working conditions, highlighting a mismatch between patients’ needs and workplace accommodation.

Moderate-to-severe disability, together with female sex, emerged as a key determinant of unemployment in IEI patients, suggesting that functional impairment—rather than disease activity alone—is associated with work exclusion. Conversely, higher educational attainment was associated with a higher likelihood of employment, supporting its role as a potential lever to reduce employment inequalities in IEI as in other chronic diseases (48). Non-employed individuals reported worse outcomes across multiple HRQoL domains, including autonomy, daily planning, leisure activities, and treatment-related distress. Collectively, these findings are consistent with evidence from chronic immune–mediated diseases, in which work participation is more closely associated with functional status and quality of life than with disease activity alone (18, 31, 32, 33).

Among employed individuals, IEI imposed a substantial burden on productivity, with nearly 40% overall work impairment. Absenteeism was primarily associated with healthcare-related time demands, whereas presenteeism and overall work impairment were mainly driven by disease burden and psychological distress. These findings highlight the need for integrated care strategies aimed at reducing disease burden, minimizing healthcare demands, and supporting mental health (31). In line with this, treatment burden, including time dedicated to therapies and management of treatment-related adverse events, also contributed to work impairment, whereas the modality of Ig administration (intravenous vs. subcutaneous) was not associated with work productivity outcomes.

Conversely, the need for immunosuppressive therapies, reflecting more severe noninfectious immune dysregulation, was also independently associated with worse work productivity, particularly in terms of presenteeism. This pattern suggests that more severe disease may not necessarily lead to increased work absence, but rather to reduced performance while working. Beyond work-related outcomes, activity impairment was similarly associated with disease burden and psychological distress, as well as with the presence of noninfectious IEI manifestations requiring immunosuppressive treatment, and was higher among women. Moreover, time dedicated to therapies was independently associated with greater activity impairment, highlighting the impact of ongoing treatment burden on daily functioning.

Notably, the route of Ig administration (subcutaneous vs. intravenous) was not associated with any dimension of work productivity or activity impairment in multivariable models. Although subcutaneous administration is often associated with greater convenience and patient satisfaction compared with the intravenous route (49, 50), the data are consistent with our previous work, showing that treatment-related quality of life is primarily influenced by patients’ attitudes rather than by treatment modality per se, and that the impact of the IgRT route of administration is mitigated when therapy is individualized (27).

In this cohort, having an IEI diagnosis alternative to PAD did not affect occupation or productivity, despite non-PAD patients showing a higher burden of healthcare-related time, including more days spent on visits and hospitalizations. However, given the small sample size of the non-PAD subgroup, these findings should be interpreted with caution and cannot be considered conclusive.

Caregivers also faced significant work-related challenges in maintaining employment and work productivity. The predominance of women reflects the well-known gendered distribution of caregiving responsibilities, with women bearing most care duties (43). Although most caregivers were employed, they reported reduced work ability, difficulties in disclosing their role, negative career impact, and unsafe workplace environments.

Caregiving imposed a substantial time burden, resulting in overall work impairment exceeding 34%, primarily driven by patient’s burden due to the IEI. Absenteeism was associated with caregiving intensity, whereas presenteeism was related to psychological distress, poorer patient health status, and child caregiving, reflecting the distinct logistical and emotional dimensions of caregiving burden. In contrast, impairment outside the workplace was mainly associated with patient disability (40). Caregivers of children experienced a higher burden, with more time and greater organizational adjustments in daily life. This translated into different work patterns, with a higher likelihood of part-time employment and a greater healthcare-related time burden, particularly due to hospitalizations. Notably, caregiving for a child was associated with reduced work performance while at work, rather than increased work absence. Overall, these findings support a dual-target approach to improving work participation in IEI, addressing both disease control in patients and psychosocial support and caregiving demands in their families.

The magnitude of work impairment observed in adults with IEI and in their caregivers is comparable with that reported in other chronic immune–mediated diseases, such as inflammatory bowel disease (51) and multiple sclerosis (52), and appears higher than that observed in rheumatoid arthritis (33). In inflammatory bowel disease, greater disease activity is associated with impaired quality of life, reduced work productivity, and increased healthcare utilization (53, 54), with pooled estimates showing overall work impairment of ∼39%, mainly driven by presenteeism (35.9%) (39). Similar patterns have been described in rheumatoid arthritis, where work impairment closely correlates with disease activity, pain, and functional limitations (33), and in multiple sclerosis, where presenteeism is strongly associated with fatigue, disability, and mental health (50). Together, these findings underscore the systemic impact of immune dysregulation ultimately translating into reduced work productivity and support the inclusion of IEI in occupational health and workplace policy frameworks.

Notably, a proportion of patients and caregivers choose not to disclose their condition, with reports of negative job consequences highlighting persistent workplace stigma. This “disclosure dilemma,” in which individuals balance the need for workplace support against the risk of adverse career outcomes, is consistent with previous literature showing that many individuals with chronic conditions avoid disclosure due to these concerns, as decisions are shaped by perceived social and occupational trade-offs (55, 56).

In Italy, all individuals with IEI are fully exempt from healthcare costs, regardless of their certified degree of disability. In our cohort, approximately half of IEI patients had formal recognition of moderate/severe disability, allowing access to additional economic benefits. This contrasts with findings from the only comparable study conducted in Japan, which reported disability certification in only 13% of patients (26). Although this suggests a good capacity of the Italian welfare system to recognize disability in IEI, access to more protective measures for workplace accommodations and caregiver-related benefits remains limited. This is particularly relevant, as such measures are intended to support job retention, preserve income stability, and prevent social vulnerability.

Study limitations included the cross-sectional design, which precluded causal inference. The exclusive inclusion of Italian participants may limit applicability to other healthcare systems and sociocultural contexts. The voluntary nature of participation introduces potential selection bias. All data were self-reported without clinical verification, although consistent with previous studies (57, 58). IEI diagnoses were self-reported and not independently verified, which may have introduced misclassification bias. Socioeconomic status and distance to healthcare facilities were not assessed, although these factors may influence both quality of life and work-related outcomes. Furthermore, the study population was predominantly composed of CVID patients, which may limit the generalizability of these findings to other IEI subtypes. Finally, patients and caregivers were not matched, precluding direct assessment of patient–caregiver interrelationships. The absence of a control group limits the comparative interpretation of the observed burden.

In conclusion, IEI exerts a dual burden on the workforce, affecting both patients and caregivers through clinical morbidity, psychological distress, and ongoing healthcare demands. Strengthening workplace support, improving recognition of functional limitations, and promoting stigma-free communication are essential to enhance job retention and quality of life. Interventions to improve workplace awareness and promote social inclusion should include flexible working arrangements, such as remote work or dedicated workspaces, adequate recovery time following treatments, and better coordination of care pathways to reduce healthcare-related demands and absenteeism. In parallel, increased awareness among employers and occupational health professionals, together with advocacy by scientific societies and patient organizations, is needed to ensure that IEIs are adequately recognized within disability certification systems (Video 1). Clinicians should also prioritize early diagnosis and timely management to prevent disability. Caregivers, whose work impairment reflects caregiving intensity, patient health status, and psychological burden, should be supported through dedicated strategies, including recognition of caregiving roles, flexible work arrangements, psychological support, and coordinated care pathways.

As survival and life expectancy improve in IEI, employment outcomes are emerging as a key social determinant of health, affecting income, daily functioning, and clinical outcomes. The integration of occupational outcomes into clinical guidelines, including their assessment using validated instruments, is essential to optimize long-term care and overall well-being of patients and their families, and to reduce their risk of social vulnerability.

## Materials and methods

### Study design and study population

Cross-sectional study to investigate unmet needs related to work activities in adults with IEI (>18 years) and in informal caregivers through an electronic survey. Participants were included if aged ≥18 years and if they reported having been diagnosed with IEI or acting as informal caregivers. Caregivers of both adult and pediatric individuals with IEI were included, with pediatric cases defined as individuals younger than 18 years. IEI diagnosis was self-reported by participants and referred to their clinical diagnosis established at specialized centers, according to ESID criteria (59).

Patients and caregivers attending Italian IEI referral centers were informed about the survey at the time of a visit or by e-mail; patients and caregivers affiliated to AIP were invited via e-mail or newsletters. Responses with incomplete employment data or without consent were excluded. Participants could access the patient- and caregiver-specific questionnaires by scanning a QR Code that directed them to separate surveys hosted on SurveyMonkey. Therefore, responses from patients and caregivers were not matched. This was an observational survey study; therefore, no randomization or blinding was performed. All participants provided informed consent. The questionnaire was anonymous; therefore, ethical approval was not required under local regulations for non-interventional studies.

### Study questionnaires

To measure work productivity and activity impairment, participants completed the following PROMs: WPAI:GH. This six-item questionnaire includes two 0–10 visual analogue scales. It generates four outcomes expressed as percentages. Higher scores indicate greater impairment. Outcomes include absenteeism (work time missed), presenteeism (impairment while working), overall work impairment, and activity impairment (impairment outside work). The first three outcomes are calculated only for employed participants. Scores were derived as previously described (33).

#### General Health Questionnaire-12 (GHQ-12)

The GHQ-12 is a self-administered, 12-item questionnaire assessing psychological distress. Answers are given on a four-point scale. When scored using the binary method (0–0–1–1), it yields a total score of 0–12. Scores ≥4 indicate risk of anxiety or depression (60, 61).

#### CVID_QoL

For individuals with IEI, we used the CVID_QoL, a disease-specific, self-administered questionnaire that was initially validated for CVID (16) and later applied to other IEIs (28, 29). Available in several languages (28, 62, 63, 64, 65), it includes 32 items, each rated on a five-point scale, with higher scores indicating greater disability. The questionnaire provides a global score (total CVID_QoL) and three domain scores: EF, RF, and gastrointestinal and skin symptoms. All scores are expressed as a percentage of the maximum possible score.

#### PedsQL inventory questionnaire

Caregivers completed the PedsQL 4.0 Parent Report (adult and child versions), a validated multidimensional instrument for HRQoL assessing physical, emotional, social, and school functioning with age-specific modules. Scores range from 0 to 100, with higher scores indicating better perceived HRQoL (66, 67).

### Data collected

Demographic variables included age, sex, educational level, and family status. Occupational history, including employment status, working hours, and job setting, was also collected. We also gathered job-related unmet needs using a structured survey developed through patients and caregivers focus groups conducted with AIP (for content, see Table S1). Clinical data included IEI diagnosis, treatments, complications, and infections in the previous year (patients only). Infections assessed over the previous year included sinusitis, otitis, bronchitis, urinary tract infections, gastroenteritis, and skin infections. Participants also reported the occurrence of severe infections, including pneumonia, meningitis, and sepsis/systemic infections. Complications of IEI included autoimmune diseases (autoimmune cytopenias, arthritis, psoriasis, and connective tissue disorders), oncohematologic conditions (lymphoma, myeloma, leukemia, and polycythemia), solid tumors, severe allergies, chronic respiratory diseases (including asthma, chronic obstructive pulmonary disease, interstitial lung disease, emphysema, and granulomatous lymphocytic interstitial lung disease), and gastrointestinal diseases (including inflammatory bowel disease, portal hypertension, hepatitis, and cholangiopathies). Treatments investigated included antibiotic, antiviral, and antifungal prophylaxis, systemic corticosteroids, DMARDs (including mycophenolate, azathioprine, cyclosporine, and methotrexate), biologic therapies (rituximab, adalimumab, abatacept, etanercept, infliximab, dupilumab, omalizumab, mepolizumab, reslizumab, benralizumab, ruxolitinib, tofacitinib, baricitinib, upadacitinib, abrocitinib, tocilizumab, secukinumab, vedolizumab, and risankizumab) chemotherapy, and other treatments as specified by participants. Patients receiving at least one of systemic corticosteroids, DMARDs, or biologic therapies were classified as having non-infectious manifestations requiring immunosuppressive treatment, as a proxy for more severe immune dysregulation. Disability status (40) and daily functional impairment (42), as certified by the INPS, were also collected.

### Statistical analysis

Continuous variables were assessed for distribution and summarized as median and IQR, or as mean and SD, as appropriate based on their distribution and the nature of the variable. In particular, WPAI outcomes and other time-related variables were reported as mean ± SD in accordance with standard reporting practices, despite their non-normal distribution. Categorical variables were expressed as frequencies and percentages. Group comparisons were performed using the nonparametric Kruskal–Wallis test or the Mann–Whitney U test for continuous variables and the chi-squared test for categorical variables. Multivariable logistic and linear regression models assessed factors associated with employment and WPAI domains. Variables were selected a priori based on clinical relevance and literature and refined through univariable analyses. Parsimonious models helped reduce overfitting. Sex was included as a covariate in multivariable models. No formal power calculation was performed; sample size was based on nationwide recruitment feasibility. Analyses used complete-case data; no imputation was applied. Demographic and clinical variables were completed due to the survey’s mandatory fields. Participants were included in the analysis only if they provided information on their employment status, as this variable was essential for the study objectives. Completion of core survey sections (demographic, clinical, and occupational data) was mandatory, resulting in no missing data for these variables. Missing data reflected partial survey completion, with item-level completion rates exceeding 90%. Given the low proportion of missing data, no imputation procedures were applied, and analyses were conducted using complete-case data. Analysis was performed using IBM SPSS Statistics version 22.0 (RRID:SCR_002865, IBM Corp.). Graphs were generated using GraphPad Prism, version 8.2.1 (RRID:SCR_002798, GraphPad Software). Statistical significance was set at P < 0.05.

### Online supplemental material

Supplementary material provides details on survey items addressing work-related difficulties and unmet needs among IEI patients and their caregivers. It includes univariate analyses of determinants of employment status, WPAI:GH dimensions in adults with IEI and caregivers, as well as quality of life by employment status and multicollinearity assessments. Additional analyses examine employment, IEI-related days lost, and work impairment by IEI diagnostic category (PAD vs. other IEIs), characteristics of patients cared for by caregivers, and associations of disability severity and daily functioning impairment with quality of life and work impairment. This article includes three supplementary figures and eight supplementary tables. Fig. S1 shows occupation and work productivity and activity impairment across IEI categories. Fig. S2 reports characteristics of patients cared for by caregivers. Fig. S3 shows disability certification and occupational burden. Table S1 shows survey content addressing job-related difficulties and unmet needs among IEI patients and caregivers. Table S2 shows determinants of employment status in IEI adults. Table S3 shows assessment of multicollinearity among variables included in the multivariable model for employment. Table S4 shows comparison of CVID_QoL items (D1–D32 and summary scales) and GHQ score between employed and non-employed IEI patients. Table S5 shows determinants (univariate analysis) of percentage of work time missed due to health (absenteeism) in 262 IEI employed patients. Table S6 shows multicollinearity assessment for multivariable WPAI models in IEI adults. Table S7 shows determinants of employment status in IEI caregivers. Table S8 lists the AIP and ImmunITA collaborator affiliations. Video 1 shows work participation and productivity in adults with inborn errors of immunity and their caregivers.

## Supplementary Material

Table S1 shows survey content addressing job-related difficulties and unmet needs among IEI patients and caregivers.

Table S2 shows determinants of employment status in IEI adults.

Table S3 shows assessment of multicollinearity among variables included in the multivariable model for employment.

Table S4 shows comparison of CVID_QoL items (D1–D32 and summary scales) and GHQ score between employed and non-employed IEI patients.

Table S5 shows determinants (univariate analysis) of percentage of work time missed due to health (absenteeism) in 262 IEI employed patients.

Table S6 shows multicollinearity assessment for multivariable WPAI models in IEI adults.

Table S7 shows determinants of employment status in IEI caregivers.

Table S8 lists AIP and ImmunITA collaborator affiliations.

## Data Availability

Data are available from the corresponding author upon reasonable request.
