# Supplementary material for: Work participation in inborn errors of immunity: The dual burden on patients and caregivers
Source: J Hum Immun. 2026 Oct 22;2(6):e20260077. doi: 10.70962/jhi.20260077 (PMC13689748; doi:10.70962/jhi.20260077)
Supplement: Supplementary file 1 [file jhi_20260077_tables1.docx]

### **Table S1.** Survey content addressing job-related difficulties and unmet needs among IEI patients and caregivers.

| **Questions/ Response options** | |
| --- | --- |
| Please select the option that best reflects your opinion/ Strongly agree – Partially agree – Not sure – Partially disagree – Strongly disagree | |
| **Patient’s version** | **Caregiver’s version** |
| I have specific accommodations to help manage my condition in the workplace (e.g., medical leave, flexible working hours, remote work, etc.). | I have specific accommodations to help manage my caregiving responsibilities in the workplace (e.g., leave for medical visits, flexible working hours, remote work, etc.). |
| My work environment is not safe/healthy for my condition (e.g., hygiene, infection prevention, cleanliness). | My work environment is not compatible with my caregiving responsibilities (e.g., lack of flexibility for medical needs or emergencies). |
| My health condition affects my ability to perform normal work activities. | My caregiving responsibilities affect my ability to perform normal work activities. |
| My health condition has had a negative impact on my career or opportunities for advancement. | My caregiving responsibilities have had a negative impact on my career or opportunities for advancement. |
| I have had to modify my job duties because of my health condition. | I have had to modify my job duties because of my caregiving responsibilities. |
| I find it difficult to talk about my health problems in the workplace. | I find it difficult to talk about my caregiving responsibilities in the workplace. |
| I have difficulty obtaining recognition of my rights as a worker with a chronic condition. | I have difficulty obtaining recognition of my rights as a caregiver of a person with a chronic condition. |
| I feel embarrassed to request sick leave or time off to manage my immunodeficiency. | I feel embarrassed to request sick leave or time off to care for my family member. |
| There is a lack of attention from my colleagues in reducing the risk of infection for me. | There is insufficient attention from my colleagues to reduce the risk of infection for me, and consequently for the family member I care for. |
| I have difficulty balancing my work activities with maintaining my well-being. | I have difficulty balancing my work activities with my caregiving responsibilities and personal well-being. |
