# Supplementary material for: Work participation in inborn errors of immunity: The dual burden on patients and caregivers
Source: J Hum Immun. 2026 Oct 22;2(6):e20260077. doi: 10.70962/jhi.20260077 (PMC13689748; doi:10.70962/jhi.20260077)
Supplement: Supplementary file 2 [file jhi_20260077_tables2.docx]

**Table S2. Determinants of Employment Status in IEI adults.** Retired patients and not-working students have been removed from the analysis to avoid bias. Continuous values were compared by the two-tailed Mann-Whitney U test. Categorical values were compared using the chi-squared test.

| **Variable** | **Employed (n=282)** | **Non-employed (n=67)** | **p-value** | **OR (95% CI)** |
| --- | --- | --- | --- | --- |
| **Education ≥ high school, n (%)** | 255/282 (87.9%) | 27/67 (45.7%) | **<0.0001** | 8.63 (4.65–16.1) |
| Sex (female), n (%) | 169/282 (59.9%) | 48/67 (71.6%) | 0.092 | 0.59 (0.33–1.05) |
| Living with others, n (%) | 245/282 (86.9%) | 60/67 (89.6%) | 0.684 | 1.29 (0.55–3.05) |
| Family member with IEI, n (%) | 29/282 (10.3%) | 6/67 (9.0%) | 0.921 | 0.86 (0.34–2.16) |
| IEI diagnosis (PAD vs other IEIs), n (%) | 256/282 (90.8%) | 57/67 (85.1%) | 0.478 | 1.77 (0.79–3.78) |
| **Disability (moderate/severe), n (%)** | 75/276 (27.3%) | 40/60 (66.7%) | **<0.0001** | 0.19 (0.10–0.35) |
| **IEI-related complications, n (%)** | 184/268 (68.7%) | 51/62 (82.3%) | **0.033** | 0.47 (0.24–0.93) |
| Autoimmune disease, n (%) | 78/282 (27.7%) | 17/67 (25.4%) | 0.723 | 1.12 (0.61–2.05) |
| Onco-hematologic disease, n (%) | 12/282 (4.3%) | 6/67 (9%) | 0.133 | 0.45 (0.16–1.26) |
| Solid tumors, n (%) | 16/282 (5.7%) | 6/67 (9%) | 0.185 | 0.61 (0.23–1.59) |
| Chronic respiratory disease, n (%) | 89/282 (31.6%) | 23/67 (34.3%) | 0.670 | 0.88 (0.49–1.57) |
| Chronic gastrointestinal disease, n (%) | 86/282 (30.5%) | 27/67 (40.3%) | 0.213 | 0.65 (0.37–1.15) |
| Noninfective manifestations on immunosuppressive treatment | 69/282 (24.5%) | 22/67 (32.8%) | 0.166 | 0.663 (0.37-1.18) |
| IgRT, n (%) | 275/282 (97.5%) | 64/67 (95.5%) | 0.530 | 1.84 (0.46–7.35) |
| SCIG, n (%) | 187/275 (68.0%) | 42/64 (65.6%) | 0.730 | 1.11 (0.61–2.01) |
| **Age at study time, median (IQR)** | **47 (37–57)** | **54 (47–61)** | **<0.0001** | — |
| **Age at IEI diagnosis, median (IQR)** | **34.5 (23–44)** | **41 (29–51.5)** | **0.006** | — |
| Infections per year, median (IQR) | 4 (1–7) | 4 (1–7) | 0.706 | — |
| GHQ positive, n (%) | 155 (61.0%) | 34 (63.0%) | 0.790 | 0.92 (0.50–1.69) |
| **CVID_QoL total, median (IQR)** | **37.50 (26.17–52.73)** | **48.05 (32.81–57.81)** | **0.010** | — |
| Emotional functioning | 42.11 (27.63–56.58) | 52.63 (34.21–63.16) | **0.007** | — |
| Gastrointestinal - skin symptoms | 31.25 (18.75–50.00) | 43.75 (18.75–56.25) | 0.152 | — |
| Relational functioning | 33.33 (16.67–50.00) | 41.67 (25.00–50.00) | **0.035** | — |

Abbreviations: IEI: Inborn Errors of Immunity; CVID: Common Variable Immunodeficiency; PAD: Predominantly antibody deficiencies; IgRT: Immunoglobulin replacement therapy; SCIG: Subcutaneous immunoglobulin; GHQ-12: General Health Questionnaire-12; CVID_QoL: Common Variable Immunodeficiency Quality of Life questionnaire; OR: Odds Ratio; CI: Confidence Interval; IQR: Interquartile Range.
