# Supplementary material for: Work participation in inborn errors of immunity: The dual burden on patients and caregivers
Source: J Hum Immun. 2026 Oct 22;2(6):e20260077. doi: 10.70962/jhi.20260077 (PMC13689748; doi:10.70962/jhi.20260077)
Supplement: Supplementary file 3 [file jhi_20260077_tables3.docx]

**Table S3. Assessment of multicollinearity among variables included in the multivariable model for employment.** Multicollinearity was assessed using variance inflation factors derived from a linear regression model including the same covariates as the multivariable logistic regression model. All VIF values were <1.2, indicating no evidence of multicollinearity.

| **Variable** | **Tolerance** | **VIF** |
| --- | --- | --- |
| Sex (female vs male) | 0.912 | 1.10 |
| Higher education (≥ high school) | 0.908 | 1.10 |
| IEI diagnosis (PAD vs other IEIs) | 0.943 | 1.06 |
| IEI-related complications | 0.782 | 1.28 |
| Disability (moderate/severe) | 0.756 | 1.32 |
| CVID_QoL total | 0.816 | 1.23 |
| Age | 0.383 | 2.61 |
| Age at IEI diagnosis | 0.397 | 2.52 |

Abbreviations: VIF, variance inflation factor; IEI, inborn errors of immunity; CVID, common variable immunodeficiency; CVID_QoL, Common Variable Immunodeficiency Quality of Life questionnaire; PAD, primary antibody deficiencies.
