# Supplementary material for: Work participation in inborn errors of immunity: The dual burden on patients and caregivers
Source: J Hum Immun. 2026 Oct 22;2(6):e20260077. doi: 10.70962/jhi.20260077 (PMC13689748; doi:10.70962/jhi.20260077)
Supplement: Supplementary file 4 [file jhi_20260077_tables4.docx]

**Table S4.** Comparison of CVID_QoL items (D1–D32 and summary scales) and GHQ score between employed and non-employed IEI patients. Participants with outside working age, including retirees and non-working students were excluded from the analysis. Values were compared by the two-tailed Mann-Whitney U.

| **Variable** | **Employed**  **Median (IQR)** | **Non-employed**  **Median (IQR)** | **p-value** |
| --- | --- | --- | --- |
| **GHQ score** | 14 (9-20) | 14.5 (8-21) | 0.900 |
| **CVID_QoL** | 0.38 (0.26–0.53) | 0.48 (0.33–0.58) | **0.010** |
| **EF (Emotional functioning)** | 0.42 (0.28–0.57) | 0.53 (0.34–0.63) | **0.007** |
| **RF (Role functioning)** | 0.33 (0.17–0.50) | 0.42 (0.25–0.50) | **0.035** |
| **GSS (GI, skin symptoms score)** | 0.31 (0.19–0.50) | 0.44 (0.19–0.56) | 0.152 |
| **CVID_QoL item** |  |  |  |
| **Q15. Autonomy** | 2.00 (0.00–2.00) | 2.00 (2.00–3.00) | **<0.001** |
| **Q10. Support needs** | 0.00 (0.00–2.00) | 1.50 (0.00–2.00) | **0.001** |
| **Q12. Treatment-related distress** | 1.00 (0.00–2.00) | 2.00 (0.00–2.00) | **0.004** |
| **Q5. Daily planning** | 2.00 (1.00–3.00) | 3.00 (2.00–3.25) | **0.005** |
| **Q25. Leisure time** | 2.00 (1.00–3.00) | 2.00 (1.00–3.00) | **0.008** |
| **Q18. Afraid to dead** | 1.00 (0.00–2.00) | 1.50 (0.00–2.00) | **0.019** |
| **Q24. Bothering by therapies** | 1.00 (0.00–2.00) | 2.00 (1.00–2.00) | **0.022** |
| **Q14. Limited by GI symptoms** | 1.00 (0.00–2.00) | 1.50 (0.00–2.00) | **0.026** |
| **Q7. Family care** | 2.00 (0.00–2.00) | 2.00 (1.00–3.00) | **0.028** |
| **Q20. Self isolation** | 1.00 (0.00–2.00) | 2.00 (1.00–2.25) | **0.030** |
| **Q3. Anger** | 2.00 (1.00–3.00) | 2.00 (1.75–3.00) | **0.045** |
| **Q9. Joint pain** | 2.00 (1.00–3.00) | 3.00 (2.00–3.00) | 0.052 |
| **Q11. Remain without medication** | 1.00 (0.00–2.00) | 2.00 (0.00–2.00) | 0.063 |
| **Q.8 Worsening** | 1.00 (0.00–2.00) | 2.00 (0.00–2.00) | 0.085 |
| **Q26. Skin problems** | 2.00 (1.00–3.00) | 2.00 (1.00–3.00) | 0.109 |
| **Q27. Relation** | 0.00 (0.00–2.00) | 1.00 (0.00–2.00) | 0.109 |
| **Q22. Feeling frail** | 2.00 (2.00–3.00) | 3.00 (2.00–3.00) | 0.114 |
| **Q32. Tiredness** | 3.00 (2.00–3.00) | 3.00 (2.75–3.00) | 0.141 |
| **Q16. Contagious** | 0.00 (0.00–2.00) | 0.00 (0.00–1.00) | 0.205 |
| **Q17. Work/study** | 2.00 (1.00–2.00) | 2.00 (1.00–3.00) | 0.213 |
| **Q28. Feeling sick** | 2.00 (1.00–3.00) | 2.00 (1.00–3.00) | 0.218 |
| **Q29. Embarrassment** | 2.00 (1.00–3.00) | 2.00 (1.00–3.00) | 0.242 |
| **Q23. Sex** | 1.00 (0.00–2.00) | 1.50 (0.00–2.00) | 0.372 |
| **Q4. GI symptoms** | 2.00 (1.00–3.00) | 2.00 (1.00–3.00) | 0.387 |
| **Q13. Afraid for future** | 2.00 (2.00–3.00) | 3.00 (2.00–3.00) | 0.428 |
| **Q1. Being sad** | 1.00 (0.00–2.00) | 2.00 (0.00–2.00) | 0.069 |
| **Q21 Getting sick** | 2.00 (2.00–3.00) | 2.00 (2.00–3.00) | 0.687 |
| **Q31. Bothering by other patients** | 0.00 (0.00–0.00) | 0.00 (0.00–1.00) | 0.740 |
| **Q6. Respiratory symptoms** | 2.00 (1.00–3.00) | 2.00 (2.00–3.00) | 0.882 |
| **Q30. Being contagious** | 2.00 (1.00–3.00) | 2.00 (1.00–3.00) | 0.910 |
| **Q2. Food** | 2.00 (1.00–3.00) | 2.00 (0.00–3.00) | 0.960 |
| **Q19. Limited by respiratory symptoms** | 1.00 (0.00–2.00) | 1.00 (0.00–2.00) | 0.997 |

Abbreviations: GHQ, General Health Questionnaire; CVID_QoL, Common Variable Immunodeficiency Quality of Life questionnaire; EF, Emotional Functioning; RF, Role Functioning; GSS, Gastrointestinal and Skin Symptoms score; IQR, interquartile range
