# Supplementary material for: Work participation in inborn errors of immunity: The dual burden on patients and caregivers
Source: J Hum Immun. 2026 Oct 22;2(6):e20260077. doi: 10.70962/jhi.20260077 (PMC13689748; doi:10.70962/jhi.20260077)
Supplement: Supplementary file 5 [file jhi_20260077_tables5.docx]

**Table S5** Determinants (Univariate Analysis) of Percentage of Work Time Missed Due to Health (absenteeism) in 262 IEI employed patients

| **Variable** | **Absenteeism,**  **r, (p value)** | | **Presenteeism,**  **r (p value)** | | **Overall Work Impairment,**  **r (p value)** | | **Activity Impairment,**  **r (p value)** | |  |
| --- | --- | --- | --- | --- | --- | --- | --- | --- | --- |
|  | **Patients** | **Caregivers** | **Patients** | **Caregivers** | **Patients** | **Caregivers** | **Patients** | **Caregivers** |  |
| **Days spent for visits** | **0.42 (0.001)** | **0.56 (<0.0001)** | **0.36 (<0.0001)** | **0.38 (0.001)** | **0.36 (<0.0001)** | **0.35 (0.003)** | **0.20 (0.001)** | 0.01 (0.979) |  |
| **Days spent for treatments** | **0.20 (0.001)** | **0.76 (<0.0001)** | **0.16 (0.007)** | **0.32 (0.013)** | **0.18 (0.004)** | **0.28 (0.028)** | **0.13 (0.028)** | 0.03 (0.879) |  |
| **Days for recovery (acute)** | 0.08 (0.188) | **0.43 (0.001)** | **0.20 (0.001)** | 0.21 (0.127) | **0.19 (0.002)** | 0.23 (0.105) | **0.18 (0.002)** | 0.10 (0.668) |  |
| **Weekly hours of caregiving** | NA | **0.40 (0.001)** | NA | **0.28 <0.0001)** | NA | **0.26 (<0.0001)** | NA | **0.0 (0.986)** |  |
| **Age** | 0.02 (0.711) | 0.22 (0.073) | 0.05 (0.392) | 0.21 (0.079) | 0.05 (0.386) | 0.20 (0.087) | 0.059 (0.386) | 0.00 (0.986) |  |
| **Age at IEI diagnosis (years)** | — (0.696) |  | — (0.263) |  | — (0.355) |  |  | — (0.355) |  |
| **Diagnostic delay (years)** | — (0.162) | —(0,7859 | — (0.991) | — (0.750) | — (0.998) | — (0.150) | — (0.192) | — (0.949) |  |
| **Sex (female)** | — (0.218) | — (0.964) | — (0.153) | — (0.162) | — (0.081) | — (0.152) | — (0.006) | — (0.507) |  |
| **Education ≥ high school** | — (0.855) | — (0.745) | — (0.348) | — (0.146) | — | — (0.338) | — | — (0.507) |  |
| **Living alone** | — (0.847) | NA | — (0.179) | NA | — (0.858) | NA | **— (0.001)** | NA |  |
| **Living with an IEI patient** | NA | — (0.947) | NA | — (0.485) | NA | — (0.510) | NA | — (0.562) |  |
| **Caring a child with IEI** | — (0.404) | — (0.994 ) | (0.192) | — (0.585 ) | — | — (0.752 ) | — | — (0.981) |  |
| **IEI diagnosis (PAD vs other IEIs)** | — (0.159) | — (0.006 ) | — (0.104) | — (0.200 ) | — | — (0.199 ) | — | — (0.167) |  |
| **Moderate/severe disability (patients)** | — (0.457) | — (0.549) | — (0.294) | — (0.356) | — (0.379) | — (0.661) | **— (0.003)** | — (0.024) |  |
| **Infections/year** | **0.20 (0.016)** | NA | **0.37 (<0.0001)** | NA | **0.338 (<0.0001)** | NA | **0.33 (<0.0001)** | NA |  |
| **IEI-complications** | **— (0.013)** | NA | **— (0.004)** | NA | **— (0.008)** | NA | — (0.148) | NA |  |
| **Noninfective manifestations on immunosuppressants** | **— (0.191)** | 0.462 | **— (<0.0001)** | 0.437 | **— (<0.0001)** | 0.716 | **— (<0.0001)** | 1.000 |  |
| **IgRT (IVIG *vs* SCIG)** | **— (0.044)** | — (0.409) | — (0.687) | — (0.867) | — (0.596) | — (0.867) | — (0.393) | — (0.079) |  |
| **GHQ positive** | **0.27 (<0.0001)** | 0.44 (0.715) | **0.55 (<0.0001)** | 0.38 (0.001) | **0.52 (<0.0001)** | 0.34 (0.004) | **0.51 (<0.0001)** | 0.55 (0.001) |  |
| **CVID_QoL_Total** | **0.33 (<0.0001)** | NA | **0.60 (<0.0001)** | NA | **0.58 (<0.0001)** | NA | **0.63 (<0.0001)** | NA |  |
| **PEDsQOL** | NA | 0.14 (0.399) | NA | 0.04 (0.813) | NA | 0.79 (0.702) | NA | 0.12 (0.503) |  |

Abbreviations: IEI: Inborn Errors of Immunity; CVID: Common Variable Immunodeficiency; PAD: Primary Antibody Deficiencies; unPAD: Unclassified Primary Antibody Deficiency; QoL: Quality of Life; HRQoL: Health-Related Quality of Life; GHQ-12: General Health Questionnaire-12; PedsQL: Pediatric Quality of Life Inventory; B: Regression coefficient; CI: Confidence Interval; OR: Odds Ratio; IgRT immunoglobulin replacement therapy, IVIG: Intravenous Immunoglobulin.
