# Supplementary material for: Work participation in inborn errors of immunity: The dual burden on patients and caregivers
Source: J Hum Immun. 2026 Oct 22;2(6):e20260077. doi: 10.70962/jhi.20260077 (PMC13689748; doi:10.70962/jhi.20260077)
Supplement: Supplementary file 6 [file jhi_20260077_tables6.docx]

**Table S6.** **Multicollinearity assessment for multivariable WPAI models in IEI adults.** Multicollinearity was assessed using variance inflation factors derived from a linear regression model including the same covariates as the multivariable logistic regression model. All VIF values were <2, indicating no evidence of multicollinearity

| **Variable** | **Absenteeism Tolerance (VIF)** | **Presenteeism Tolerance (VIF)** | **Overall work impairment Tolerance (VIF)** | **Activity impairment Tolerance (VIF)** |
| --- | --- | --- | --- | --- |
| **Adults with IEI** |  |  |  |  |
| CVID QoL score | 0.535 (1.87) | 0.535 (1.87) | 0.535 (1.87) | 0.524 (1.91) |
| GHQ positive | 0.641 (1.56) | 0.645 (1.55) | 0.645 (1.55) | 0.645 (1.55) |
| Days for visits/exams | 0.813 (1.23) | 0.813 (1.23) | 0.813 (1.23) | 0.820 (1.22) |
| Days for therapies | 0.893 (1.12) | 0.893 (1.12) | 0.893 (1.12) | 0.870 (1.15) |
| Age | 0.926 (1.08) | 0.926 (1.08) | 0.926 (1.08) | 0.926 (1.08) |
| Sex | 0.943 (1.06) | 0.943 (1.06) | 0.943 (1.06) | 0.952 (1.05) |
| IEI diagnosis (PAD vs others) | 0.885 (1.13) | 0.885 (1.13) | 0.885 (1.13) | 0.926 (1.08) |
| IgRT (SCIG vs IVIG) | 0.870 (1.15) | 0.870 (1.15) | 0.870 (1.15) | 0.926 (1.08) |
| Immunosuppressive therapy | 0.901 (1.11) | 0.901 (1.11) | 0.901 (1.11) | 0.917 (1.09) |
|  |  |  |  |  |
| **IEI caregivers** |  |  |  |  |
| GHQ (positive) | 0.856 (1.169) | 0.844 (1.184) | 0.844 (1.184) | 0.786 (1.273) |
| Weekly care hours | 0.819 (1.221) | 0.849 (1.178) | 0.849 (1.178) | 0.970 (1.031) |
| PedsQL total score | 0.805 (1.242) | 0.805 (1.242) | 0.805 (1.242) | — |
| Age | 0.925 (1.082) | 0.922 (1.084) | 0.922 (1.084) | — |
| Caring for a child | 0.792 (1.263) | 0.826 (1.211) | 0.826 (1.211) | — |
| Patient’s disability, (moderate/severe) | — | — | — | 0.767 (1.304) |

Abbreviations: VIF, variance inflation factor; IEI, inborn errors of immunity; CVID_QoL, Common Variable Immunodeficiency Quality of Life questionnaire, PAD: Primary Antibody Deficiencies.
