# Supplementary material for: Work participation in inborn errors of immunity: The dual burden on patients and caregivers
Source: J Hum Immun. 2026 Oct 22;2(6):e20260077. doi: 10.70962/jhi.20260077 (PMC13689748; doi:10.70962/jhi.20260077)
Supplement: Supplementary file 7 [file jhi_20260077_tables7.docx]

**Table S7.** Determinants of Employment Status in IEI-caregivers. Retired caregivers and not-working students have been removed from the analysis to avoid bias. Continuous values were compared by the two-tailed Mann-Whitney U test. Categorical values were compared using the chi-squared test.

| **Variable** | **Employed (n=77)** | **Non-employed (n=15)** | **p-value** | **OR (95% CI)** |
| --- | --- | --- | --- | --- |
| **High education level, n (%)** | 75/77 (97.4%) | 10/15 (66.7%) | 0.001 | 18.75 (3.20–109.81) |
| **Sex (female), n (%)** | 55/76 (72.4%) | 14/15 (93.3%) | 0.083 | 0.19 (0.02–1.51) |
| **IEI diagnosis (PAD), n (%)** | 55/76 (72.4%) | 6/13 (46.2%) | 0.102 | 3.06 (0.92–10.20) |
| **Cohabiting with an IEI-affected subject, n (%)** | 64/77 (83.1%) | 11/15 (73.3%) | 0.151 | 0.41 (0.12–1.41) |
| **IEI diagnosis <10 years, n (%)** | 22/58 (37.9%) | 6/25 (24.0%) | 0.312 | 2.14 (0.41–11.23) |
| **Caregiver age (years), median (IQR)** | 48 (41–57) | 47 (44–58) | 0.564 | — |
| **Age at IEI diagnosis (years), median (IQR)** | 39 (30–47) | 39 (32–48) | 0.105 | — |
| **IEI relative current age (years), median (IQR)** | 20 (4–40) | 26 (12–52) | 0.829 | — |
| **Caregiving for a child <18 years, n (%)** | 20/77 | 4/15 | 1.0000 | 0.96 (0.27–3.35) |
| **Weekly hours of caregiving, median (IQR)** | 20 (4-20) | 42 (20-130) | 0.105 | — |
| **Disability (moderate-severe), n (%)** | 35/70 (50%) | 10/14 (71.4) | 0.180 | 0.40 (0.12-1.40) |
| **IgRT, n (%)** | 69/77 (89.6%) | 12/15 (80.0%) | 0.379 | 2.16 (0.50–9.32) |
| **SCIG, n (%)** | 43/69 (62.3%) | 6/12 (50.0%) | 0.531 | 2.35 (0.76–7.28) |
| **Immunosuppressive therapy** | 18/77 | 3/15 | 1.000 | 1.22 (0.31–4.80) |
| **GHQ positive, n (%)** | 44/71 (62.0%) | 9/13 (69.2%) | 0.754 | 0.72 (0.20–2.58) |
| **PedsQL total, median (IQR)** | 69.02 (49.63–77.17) | 65.76 (47.28–82.07) | 0.943 | — |
| **Physical functioning (PF), median (IQR)** | 70.31 (28.13–87.50) | 71.88 (43.75–92.19) | 0.578 | — |
| **Emotional functioning (EF), median (IQR)** | 60.00 (42.50–77.50) | 55.00 (37.50–72.50) | 0.544 | — |
| **Social functioning (SF), median (IQR)** | 82.50 (60.00–97.50) | 75.00 (57.50–90.00) | 0.324 | — |
| **School/work functioning, median (IQR)** | 65.00 (37.50–85.00) | 62.50 (45.00–90.00) | 0.797 | — |

Abbreviations IEI, inborn errors of immunity; intravenous immunoglobulin; SCIG, subcutaneous immunoglobulin; HSCT, hematopoietic stem cell transplantation; GT, gene therapy; GHQ, General Health Questionnaire; PedsQL, Pediatric Quality of Life Inventory; PF, physical functioning; EF, emotional functioning; SF, social functioning; SCH/WRK, school/work functioning; GI, gastrointestinal; OR, odds ratio; CI, confidence interval; IQR, interquartile range; PID, primary immunodeficiency.
