# Supplementary material for: Work participation in inborn errors of immunity: The dual burden on patients and caregivers
Source: J Hum Immun. 2026 Oct 22;2(6):e20260077. doi: 10.70962/jhi.20260077 (PMC13689748; doi:10.70962/jhi.20260077)
Supplement: Supplementary file 8 [file jhi_20260077_tables8.docx]

**AIP collaborators:** Alessandro Segato, [alessandro.segato@aip-it.org](mailto:alessandro.segato@aip-it.org), AIP APS Associazione Italiana Immunodeficienze Primitive

Filippo Cristoferi, [filippo.cristoferi@gmail.com](mailto:filippo.cristoferi@gmail.com), AIP APS Associazione Italiana Immunodeficienze Primitive

Silvia Casati, [comunicazione@aip-it.org](mailto:comunicazione@aip-it.org), Associazione Italiana Immunodeficienze Primitive.

**ImmunITA collaborators:** Pietro Andrea Accardo, [p.accardo@villasofia.it](mailto:p.accardo@villasofia.it), UOS Immunologia, Azienda Ospedaliera Villa Sofia-Cervello, Palermo, Italy

Rosa Angarano, [rangarano.emat@gmail.com](mailto:rangarano.emat@gmail.com), UOC Oncoematologia Pediatrica, Azienda Ospedaliera Universitaria Policlinico Di Bari

Federica Barzaghi, [barzaghi.federica@hsr.it](mailto:barzaghi.federica@hsr.it) San Raffaele Telethon Institute for Gene Therapy and Pediatric Immunohematology and Bone Marrow Transplantation Unit, IRCCS San Raffaele Scientific Institute, Milan, Italy

Caterina Cancrini, [cancrini@med.uniroma2.it](mailto:cancrini@med.uniroma2.it), Ospedale Bambino Gesù

Clementina Canessa, [clementina.canessa@meyer.it](mailto:clementina.canessa@meyer.it), SOC Clinica Pediatrica-Immunologia Meyer Children's Hospital IRCCS

Francesca Carfora, [francesca.carfora@uniroma1.it](mailto:francesca.carfora@uniroma1.it), Department of Transitional and Precision Medicine, Sapienza University of Rome, Viale dell'Università 37, 00185 Rome

Maria Carrabba, [maria.carrabba@gmail.com](mailto:maria.carrabba@gmail.com), Fondazione IRCCS Ca' Granda Ospedale Maggiore Policlinico

Maria Pia Cicalese, cicalese.mariapia@hsr.it, San Raffaele Telethon Institute for Gene Therapy and Pediatric Immunohematology and Bone Marrow Transplantation Unit, IRCCS San Raffaele Scientific Institute, Milan, Italy, Vita-Salute San Raffaele University, Milan, Italy

Francesco Cinetto, [francesco.cinetto@unipd.it](mailto:francesco.cinetto@unipd.it), Rare Diseases Referral Center, Internal Medicine 1, Ca' Foncello Hospital, AULSS2 Marca Trevigiana, Treviso, Italy, Department of Medicine - DIMED, University of Padova, Padua, Italy

Maria Giovanna Danieli, [m.g.danieli@univpm.it](mailto:m.g.danieli@univpm.it), SOS Immunologia delle Malattie rare e dei Trapianti, AOU delle Marche, Ancona, Italy, Dipartimento di Scienze Cliniche e Molecolari, Università Politecnica delle Marche, 60126 Ancona, Italy

Giulia Di Colo, [dicolo.giulia@hsr.it](mailto:dicolo.giulia@hsr.it), IRCCS Ospedale San Raffaele Milano

Giovanna Fabio, [giovanna.fabio@unimi.it](mailto:giovanna.fabio@unimi.it), Fondazione IRCCS Ca' Granda Ospedale Maggiore Policlinico

Andrea Finocchi, [andrea.finocchi@opbg.net](mailto:andrea.finocchi@opbg.net), Department of System Medicine, University Tor Vergata, Unit of Clinical Immunology and Vaccinology, IRCSS Ospedale Pediatrico Bambino Gesù

Giuliana Giardino, [giuliana.giardino@unina.it](mailto:giuliana.giardino@unina.it), Department of Translational Medical Science

Giuseppe Ienopoli, [giuseppeienopoli10@gmail.com](mailto:giuseppeienopoli10@gmail.com), Allergy and Immunology, ASL CN1, 12100, Cuneo, Italy

Lorenzo Lodi, [lorenzo.lodi@meyer.it](mailto:lorenzo.lodi@meyer.it), Immunology Unit, Meyer Children’s Hospital IRCCS, Florence, Italy, NEUROFARBA Department, University of Florence, Florence, Italy

Baldassarre Martire, [baldassarre.martire@aslbat.it](mailto:baldassarre.martire@aslbat.it), Unit of Pediatrics and Neonatology, Maternal-Infant Department, "Monsignor A.R. Dimiccoli" Hospital, 70051 Barletta, Italy

Silvia Ricci, [Silvia.ricci@unifi.it](mailto:Silvia.ricci@unifi.it), Meyer Children’s Hospital IRCCS

Beatrice Rivalta, Unit of Clinical Immunology and Vaccinology, Ospedale Pediatrico Bambino Gesù

Riccardo Scarpa, [riccardo.scarpa@unipd.it](mailto:riccardo.scarpa@unipd.it), Rare Diseases Referral Center - UOC Internal Medicine 1 - AULSS2 Department of Medicine - DIMED University of Padova

Serena Serafino, [serafinoserena@gmail.com](mailto:serafinoserena@gmail.com), Fondazione IRCCS Cà Granda Ospedale Maggiore Policlinico di Milano

Germano Sardella, [germano.sardella@uniroma1.it](mailto:germano.sardella@uniroma1.it), Department of Transitional and Precision Medicine, Sapienza University of Rome, Viale dell'Università 37, 00185 Rome, Italy, Pediatric Immunopathology and Allergology Unit, Policlinico Tor Vergata, University of Rome Tor Vergata, 00133 Rome, Italy

Marta Chiara Sircana, [martachiara.si@gmail.com](mailto:martachiara.si@gmail.com), Department of Medical and Surgical Experimental Sciences, Universita of Sassari, Sassari, Italy

Emanuele Vivarelli, [emanuele.vivarelli@unifi.it](mailto:emanuele.vivarelli@unifi.it), Immunoallergology Unit, Careggi University Hospital, Florence, Italy.
